# Extracting standard error from the 95% confidence interval of a survival

**DOI:** 10.1371/journal.pone.0355291

**Published:** 2026-08-04

**Authors:** Atchiman Marilyn Ello, Anne Lübbeke, Christophe Combescure

**Affiliations:** 1 Department of Community Health and Medicine, University of Geneva, Geneva, Switzerland; 2 Division of Orthopaedic Surgery and Traumatology, Department of Surgery, Geneva University Hospitals and University of Geneva, Geneva, Switzerland; 3 Nuffield Department of Orthopaedics, Rheumatology and Musculoskeletal Sciences, University of Oxford, Oxford, United Kingdom; 4 Clinical Research Centre, Geneva University Hospitals, Geneva, Switzerland; Indiana University Indianapolis, UNITED STATES OF AMERICA

## Abstract

Extracting the standard error of survival from published 95% confidence intervals (95%CI) may be needed for meta-analyses. For the extraction, the transformation used to compute the 95%CI must be known. When that transformation is not stated in publications, an assumption must be made about it. However, an assessment of the impact of an incorrect assumption and a procedure to identify the correct transformation are lacking. To address this, we propose an approach aiming at identifying the transformation used to compute the 95%CI of the survival and enhancing the extraction of the standard error. This approach, named “Logarithm of the Relative Asymmetry” (LRA), is based on the asymmetry of the reported 95%CI. In this study, we assess the extraction error, which is the difference between extracted and true standard errors, and the performance of the LRA approach. The impact of the extraction error and the LRA approach on meta-analyses is also assessed. This study shows that an incorrect assumption about the transformation used to compute the 95%CI of the survival causes, in most cases, an overestimation of the standard error. This overestimation worsens as the survival approaches 0 or 1 or as the sample size decreases, and it propagates into the meta-analysis results. The LRA approach correctly identifies the transformation, yet its performance may be negatively impacted by the number of decimal places used to report the survival and its 95%CI or when the lower or upper bound of the reported 95%CI is exactly 0 or 1 respectively. Overall, the LRA approach is suitable for avoiding important extraction errors and mitigates their impact on meta-analyses results. Thus, we recommend this approach to identify the transformation used to compute the 95%CI of the survival.

## Introduction

Published information cannot always be directly reused to conduct aggregated data analyses and may require further processing. The processing step is essential for meta-analyses consisting of a quantitative synthesis of estimates coming from diverse sources (articles, reports, etc.) and in the computation of a pooled estimate. Several methods have been developed for this purpose. Hozo et al. [1] developed a method to obtain the mean and the variance of continuous outcomes from the reported median, range and sample size of a study. In studies reporting survival outcomes, the reported results are often Kaplan-Meier survival curves or survival estimates at specific timepoints with their 95% confidence intervals (95%CIs). Thus, Guyot et al. [2] proposed an approach to derive an approximation of the original individual patient data from the Kaplan-Meier survival curve, whereas Parmar et al. [3] focused on the extraction of the hazard-ratio and its variance from published results. For meta-analyses of survival estimates at a given time point, the standard errors of the said estimates are needed to compute the pooled survival. When only the survival and its 95%CI are given, the above-mentioned methods are unsuitable for the extraction of the standard error. Knowing that this standard error can be extracted from the 95%CI of the survival, we aim to develop a method based on said interval.

Extracting the standard error from the 95%CI of the survival raises some methodological issues. When doing the extraction, the method used to compute the 95%CI must be taken into account but this method is not systematically mentioned in publications. Thus, an assumption must be made about the method, but an incorrect assumption can lead to an erroneous value of the extracted standard error. Although all methods of computing the 95%CI of the survival are asymptotically equivalent, they may yield different 95%CIs especially regarding the asymmetry level when the sample size is small [4]. The asymmetry could be valuable information for identifying the method of computation of the 95%CI and thereby for improving the extraction of the standard error. Another issue that could impact the extraction of the standard error is the rounding of the reported survival and its 95%CI, and the number of decimal places varies across publications. These estimates are sometimes reported with four, three or two decimal places which corresponds to percentages reported with two, one or no decimal places respectively. The level of precision of the reported numbers could impact the asymmetry and may invalidate its use as an indicator of the transformation used to compute the 95%CI of the survival.

In this paper, we first present the transformation-based method of computation of the 95%CI of the survival and the general procedure for extracting the standard error from 95%CIs reported in publications. Then, we study the extraction error as a function of the transformation used to compute the survival 95%CI and the assumption made about it. We consider different rounding schemes for the reported estimates. We focus on identity, logarithm, complementary log-log, logit and arcsine transformations, as they are the most commonly available in statistical software. We then propose a novel approach to determine the transformation used to compute the reported 95%CI based on asymmetry and evaluate its performance for different rounding schemes. Finally, using a simulation study and an illustrative example, we assess the impact of an extraction error and the performance of the novel approach we propose on the results of meta-analysis.

## Method

### Extraction error of the standard error of the survival

Assuming that the survival estimator transformed with a one-to-one function f is normally distributed [5], the 95%CI of the survival $\hat{S}$ (the index of the time is omitted for readability purposes) is obtained as follows:


$$\begin{array}{c} f^{\;-\text{1}}\left(f\left(\hat{S}\right)\pm Z_{\text{0.975}}\;SE_{f\left(\hat{S}\right)}\right) \end{array}$$ (1)

with Z_0.975_ the 0.975-quantile of a standard normal distribution, $SE_{f\left(\hat{S}\right)}$ the standard error of $f\left(\hat{S}\right)$, and $f^{\;-\text{1}}$ the inverse off. The expressions of $f^{\;-\text{1}}$ and $SE_{f\left(\hat{S}\right)}$ in function of $SE_{\hat{S}}$ obtained with the delta-method are summarised in S1 Table forvarious transformations. The computation of the 95%CI (equation (1)) can lead to values lower than 0 or greater than 1 for the lower and upper bounds respectively. In these cases, the 95%CI bounds are truncated, that is, the lower bound is reported as 0 or the upper bound as 1. The lower bound is truncated when the survival is close to 0 and the 95%CI is computed with the identity transformation. The upper bound is truncated when the survival is close to 1 and the 95%CI is computed with either the logarithm or the identity transformation.

From equation (1), $SE_{f\left(\hat{S}\right)}$ can be derived from the lower and upper bounds of the 95%CI, respectively denoted $UB_{f}$and $LB_{f}$: $SE_{f\left(\hat{S}\right)}=\left|f\left(UB_{f}\right)-f\left(LB_{f}\right)\right|/\;(\text{2}{\;Z}_{\text{0}.\text{9}\text{7}\text{5}})$. Since the 95%CI of $f\left(\hat{S}\right)$ is symmetric, $SE_{f\left(\hat{S}\right)}$ can also be derivedusing only one bound: $SE_{f\left(\hat{S}\right)}=\left|f\left(UB_{f}\right)-f\left(\hat{S}\right)\right|/{\;Z}_{\text{0}.\text{9}\text{7}\text{5}}$ and analogously using the lower bound. However, thiscomputation requires the transformation  f  to be known. When this is not the case, the analyst assumes, potentially erroneously, that a transformation  g  was used to compute the 95%CI and hence the following quantity is extracted:


$$\begin{array}{c} SE_{g\left(\hat{S}\right)}^{extracted}=\frac{\left|g\left(UB_{f}\right)-g\left(LB_{f}\right)\right|}{\text{2}\;Z_{\text{0}.\text{9}\text{7}\text{5}}} \end{array}$$ (2)

If  g  is different from f, that is the assumption made by the analyst is incorrect, the extracted value $SE_{g\left(\hat{S}\right)}^{extracted}$ is wronglytaken as the standard error of the *g*-transformed survival.

We define the extraction error, denoted *EE*, by the relative difference in percentage between $SE_{g\left(\hat{S}\right)}^{extracted}$ converted on the natural scale of survival, and $SE_{\hat{S}}$ (the correct standard error of the survival):


$$\begin{array}{c} EE=\text{1}\text{0}\text{0}\cdot \frac{h\left(SE_{g\left(\hat{S}\right)}^{extracted}\right)-{SE}_{\hat{S}}}{{SE}_{\hat{S}}} \end{array}$$ (3)

with h the function converting a given standard error into what we believe to be the standard of the survival $\hat{S}$ using the delta method (S1 Table).

### Identification of the transformation f

#### General case.

Depending on the transformation  f  used for the computation, the resulting 95%CIs can be different, especially in terms of symmetry. For example, the 95%CI is symmetric when  f  is the identity transformation. When  f  is the logarithm, the upper bound ends up further from the estimate than the lower bound because the inverse function (exponential) is increasing. Regardless of the chosen transformation, the 95%CI of *f*(Ŝ) is always symmetric. Thus, a practical way to identify the transformation  f  is to find the transformation  g  yielding the less asymmetric g*-*transformed 95%CI. To make the index symmetric around 0, we suggest choosing the transformation that minimises the absolute value of the logarithm of the relative asymmetry (LRA):


$$\begin{array}{c} g^{LRA}=\text{arg}\underset{g}{\text{min}}\left|\text{ln}(\frac{g\left(UB_{f}\right)-g\left(\hat{S}\right)}{g\left(\hat{S}\right)-g\left(LB_{f}\right)})\right| \end{array}$$ (4)

We limit ourselves to the most commonly available transformations in statistical software: identity, logarithm, complementary log-log, logit and arcsine. Additional theoretical justification of the LRA approach can be found in the S1 File.

#### Special cases of $UB_{f}=1$ and$LB_{f}=0$.

As mentioned above, some transformations used to compute the 95%CI of the survival can lead to $UB_{f}\;$ or $LB_{f}\;$ being reported as 1 or 0 respectively. Also, the rounding precision can lead to $UB_{f}\;$ or $LB_{f}\;$ being reported as 1 or 0 respectively. In these cases, the LRA approach described above cannot be applied. Therefore, we extend it to the truncated and rounded to 0 or 1 cases by trying to recover the actual value of the bound knowing that when the 95%CI of a survival $\hat{S}$ is computed with the transformation f, $f\left(UB_{f}\right)-\;f\left(\hat{S}\right)=\;f\left(\hat{S}\right)-\;f\left(LB_{f}\right)$. For readability purposes in this section, we will refer to upper bounds equal to 1 and lower bounds equal to 0 as truncated even if it is due to rounding.

The algorithm of the LRA approach for the truncated cases is the following:

1. Transform the survival $\hat{S}$ and untruncated bound with candidate transformation  g  within the scope of the five transformations considered;
2. Reconstruct the potential real value of the truncated bound as:

$$\begin{array}{c} \text{2}g\left(\hat{S}\right)-\;g(\text{untruncated bound}) \end{array}$$

This is derived from $f\left(UB_{f}\right)-\;f\left(\hat{S}\right)=\;f\left(\hat{S}\right)-\;f\left(LB_{f}\right)$;

3. Put the reconstructed bound to 0 or 1 if needed or round it to the same number of decimal places as the reported survival;
4. Repeat steps 1–3 for all transformations  g  within the set considered,
5. $g^{LRA}$ is the transformation  g  leading to a truncated value of the bound and is then used to extract the standard error:$\frac{\left|g^{LRA}\left(\hat{S}\right)-g^{LRA}\left(LB_{f}\right)\right|}{\text{2}\;Z_{\text{0}.\text{9}\text{7}\text{5}}}$ when the upper bound is truncated and $\frac{\left|g^{LRA}\left(UB_{f}\right)-g^{LRA}\left(\hat{S}\right)\right|}{\text{2}\;Z_{\text{0}.\text{9}\text{7}\text{5}}}$ when the lower bound is truncated.

If more than one transformation leads to a truncated bound, i.e., multiple compatibilities, we compute as many $SE_{g\left(\hat{S}\right)}^{extracted}$as the number of transformations yielding a truncated bound and average them to obtain the extracted standard resulting from using the LRA approach.

If no transformation leads to a truncated bound, i.e., no compatibility, we choose $g^{LRA}=id$ if the lower bound is truncated and $g^{LRA}=ln$ if the upper bound is truncated. Indeed, when the lower bound is truncated, applying the LRA approach to 95%CIs computed using the identity transformation leads, more than for any other transformation, to cases where the LRA approach yields no compatibility. The same logic applies to the logarithm when the upper is truncated. The process and results leading to this decision rule can be found in S2 File.

### Assessment of the extraction error for a single survival

The extraction error is assessed deterministically for all combinations of transformations  f  and  g  within the set of identity, logarithm, complementary log-log, logit and arcsine transformations. The extraction error for $g=g^{LRA}$ is also assessed. For a given survival value $\hat{S}$ and effective sample size *n’*, the standard error of $\hat{S}$ is obtained by $\sqrt{\frac{\hat{S}\left(\text{1}-\hat{S}\right)}{n^{\prime }}}$. The effective sample size is the sample size an uncensored survival analysis would require to achieve the same standard error for a given survival estimate as a survival analysis with censoring. Thus, in our assessment, we use the effective sample size to have comprehensive scenarios. To help with the interpretation of the results, the correspondence between effective sample size and sample size for different censoring rates and survival values can be found in the S3 File. The 95%CIs are obtained using the relationships shown in S1 Table for the different transformations. The standard errors are then extracted using equation (2) and the extraction error is computed as defined in equation (3). The following conditions are investigated:

- effective sample sizes *n’* (from 10 to 1000 in increments of 10)
- survival from 0.001 to 0.999 compatible with the effective sample sizes considered
- 95%CI reported rounded to four, three or two decimal places corresponding to two, one or no decimal places respectively when expressed as percentages.

An additional assessment of the extraction error yielded by the LRA approach is done for cases where the 95%CI is computed using bootstrap percentile and rounded to three decimal places (S4 File).

All computations are performed with the software R version 4.6.0 (2026-04-24 ucrt) [6].

### Assessment of the extraction error in meta-analyses of survival

The impact of an incorrect extraction on the results of a meta-analysis of survival is assessed with a simulation study. Individual survival times are randomly generated for 10 studies assuming an exponential survival model with a constant hazard of 0.05. The survival at a specific time point is assessed in each study with Kaplan-Meier estimator and the 95%CI is computed assuming a transformation f. The standard error is extracted from the 95%CI rounded to three decimal places and assuming a transformation  g  for all studies. The survival estimates rounded to three decimal places are then pooled in a meta-analysis. The average absolute difference between the pooled estimates obtained assuming the correct transformation, i.e.,  g=f, and assuming an incorrect transformation, i.e., g≠f, is computed. We refer to this difference as the difference with the correct pooled survival. The average relative difference in percentage between the standard errors of the pooled survival obtained assuming g=f and the standard error of the pooled survival assuming g≠f are also computed. We refer to this difference as the difference in standard error (SE). All combinations of transformations f and g are within the set of identity, logarithm, complementary log-log, logit and arcsine functions. Additionally, the performance of the LRA approach is assessed by taking $g=g^{LRA}$ (equation (4)).

Various settings were assessed:

- value of the survival parameter
- sample size of each study included in the meta-analysis: either 30, 50, 100, 150 or 300
- censoring rate: an exponential distribution of the censoring times is assumed with a hazard such that the censoring rate is 0, 25 or 50%. Other combinations of censoring and survival are assessed and the results are shown in S3 File.
- level of heterogeneity between studies:
  - no heterogeneity: the parameter value of the survival is identical in all studies and survival estimates are pooled assuming a fixed effects model (inverse of variance method). In this setting, the assessed survival parameter is 0.25, 0.50 or 0.75;
  - presence of heterogeneity: a large random effect (standard deviation = 0.8) is added on the hazard of the exponential survival model. Survivals are then pooled assuming a random effects model (REML estimator of the between-studies variability). In this setting, the assessed survival parameter is 0.25, 0.50 or 0.75.

For each setting, meta-analyses are performed by combining the survival estimates on all three of the complementary log-log, the log and the logit scales. Also, 5000 meta-analyses are conducted for each setting. To enable the comparison, meta-analyses results (pooled estimates and standard errors) are converted to the natural scale of survival. The package metafor [7] version 4.6−0 for R is used for the models with random effects. When the generated survival was 0 or 1 in a study, a continuity correction of 0.5 was applied. The procedures of data generation and analyses are detailed in S5 File.

### Artificial intelligence tools and technologies

All R scripts generating data for this work were written by the authors. For the script of the assessment of the LRA approach on bootstrap-generated-95%CIs, the one creating the function implementing the LRA approach and the ones assessing meta-analysis results for other combinations of survival and censoring times distributions, Claude Sonnet 4.6 and Microsoft 365 Copilot (version 2.20260623.37.0) were used to optimise the author-written code to reduce the computing time. All AI-generated code was reviewed, verified line by line and eventually modified and corrected by the authors.

## Results

### Extraction error

**Fig 1:**
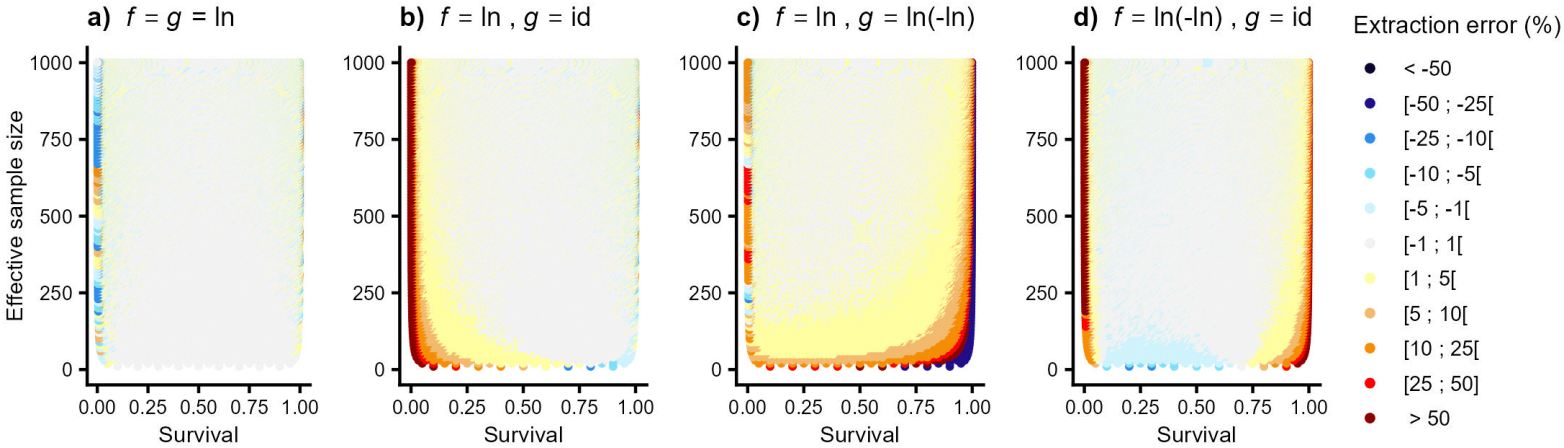
Extraction error for survival and 95%CIs reported with three decimal places.

Fig 1 shows that the choice of transformation  g  used for the extraction of the standard error has an impact on the extracted value. For readability, the figure presents four combinations of f and  g  showing four different patterns of extraction error as a function of the effective sample size. Fig 1 presents the setting in which the survival and 95%CI are reported rounded to three decimal places. The extraction error for the other combinations of f and g, and other rounding schemes can be found in S6 File.

When  g  is the same as the transformation that has been used to compute the 95%CI, i.e.,  f=g, the *EE* is below ±1% for most values of the survival and effective sample sizes (Fig 1a). In this setting, the cases where *the EE* is higher than ±1% reveal the impact of the truncation and the reporting precision of the survival and its 95%CI on the extracted standard errors: the fewer decimal places reported, the higher the *EE* (S6 File).

When f≠g, the magnitude and occurrence of *EE* tend to be more important for survival values close to 0 or 1 and smaller effective sample sizes. For instance, when f=ln  and g=id (Fig 1b), *EE* occurs when the survival is close to 0 whatever the effective sample size and when the survival is between 0.5 and 0.75 for small effective sample sizes. The magnitude of *EE* in these cases falls between 1% and 5% and can be above 50% for the smallest values of the survival. This pattern of error is observed for other combinations of  f  and  g  (S6 File). When f=ln and g=ln(−ln) (Fig 1c), most *EEs* arise when the survival is close to 0 or 1 or for effective sample sizes lower than approximately 250. In most cases, the magnitude of the *EE* falls between 1% and 5% but can be above 25% or up to −50% for the smallest effective sample sizes or highest survival values. This pattern is observed for other combinations of  f  and  g  (S6 File). The EE can be negative as it is the case when f=ln(−ln) and g=id (Fig 1d). This occurs mainly for small effective sample sizes.

### Performance of the LRA approach

**Fig 2:**
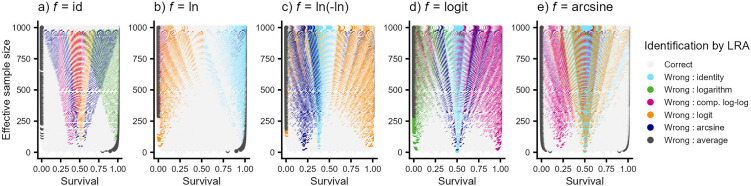
Performance of the LRA approach in identifying *f* for a survival and 95%CI reported with three decimal places.

The LRA approach is, in most cases, able to identify the transformation  f  used to compute the 95%CI of the survival when the latter and the survival are reported rounded to three decimal places as shown in Fig 2. An incorrect identification of the transformation  f  occurs when the survival is around 0.20, 0.40 or 0.50, close to 0 or 1 depending on  f  or for some truncated 95%CIs. The range of survival values for which there is a misidentification increases as the effective sample size increases. The more decimal places reported for the survival and its 95%CI, the better the performance of the LRA approach (S7 File).

**Fig 3:**
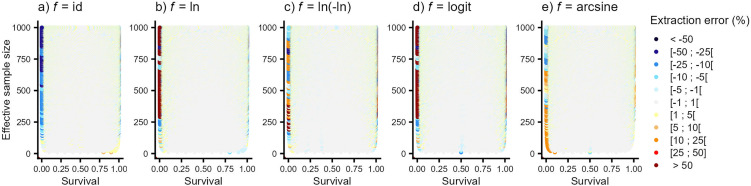
Extraction error when LRA approach is used to identify *f* for a survival and 95%CI reported with three decimal places.

A misidentification of the transformation  f  by the LRA approach does not systematically result in an important extraction error. Fig 3 presents the EE resulting from using the LRA approach to identify the function used to compute the 95%CI of the survival (rounding to three decimal places). Overall, this EE is similar to the one resulting from the use of the correct transformation, i.e., g=f (Fig 1a). This result is observed for all censoring rates and number of decimal places reported for the survival and its 95%CI (S7 File).

### Meta-analyses simulations

**Fig 4:**
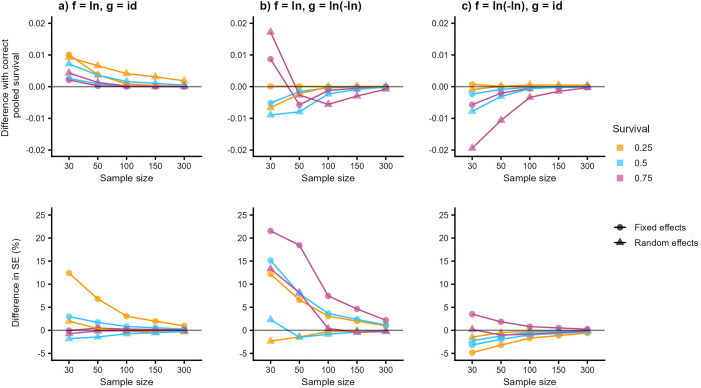
Difference with correct pooled survival and difference in standard error for a 25% censoring rate.

For readability, Fig 4 presents the results for the same combinations of  f  and  g  presented in the sections above for a 25% censoring rate. Fig 4 top panels present the difference with the correct pooled survival. A positive difference with the correct pooled survival means an overestimation. The difference with the correct pooled survival for the other combinations can be found in the supporting information (S8 File). For both fixed and random effects meta-analyses, the difference with the correct pooled survival increases as the sample size decreases or as the censoring rate increases (S8 File). Also, the closer the survival parameter value is to 0 or 1, the more important the difference with the correct pooled survival is. For instance, in the random effects setting, for a survival parameter of 0.25 and a sample size equal to 30, the difference can be up to 4% of the survival parameter value when f=ln and g=id (Fig 4a top panel).

Fig 4 bottom panels present the difference in standard error. A positive difference in standard error means an overestimation. The difference in standard error for the other combinations can be found in the supporting information (S8 File). For both fixed and random effects meta-analyses, the difference in standard error increases as the sample size of studies included in the meta-analysis decreases or as the censoring rate increases (S8 File). Also, the closer the survival parameter value is to 0 or 1, the more important the difference in standard error. For instance, the difference can be up to 20% for a sample size equal to 30 and a survival of 0.75 (Fig 4b, bottom panel). The extraction error has a more important impact on the standard error of the pooled survival than on the pooled survival itself.

**Fig 5:**
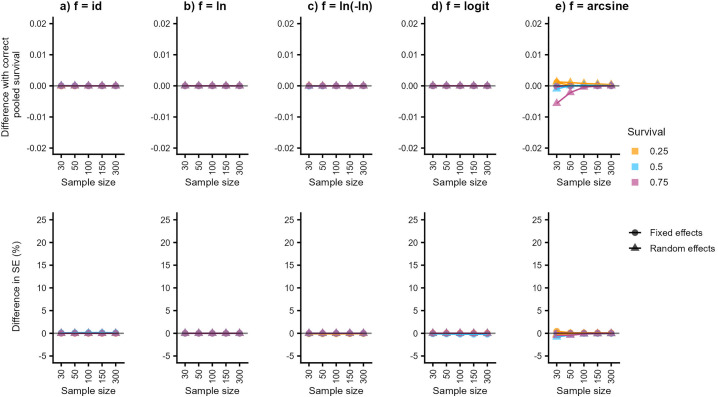
Difference with the correct pooled survival and difference in standard error when the LRA approach is used. The censoring rate is 25%.

Fig 5 top panels show the difference between the correct pooled survival and the pooled survival obtained when the LRA approach is used. Fig 5 bottom panels show the difference between the standard error of the correct pooled survival and the standard error of the pooled survival obtained when the LRA approach is used. In fixed and random effects settings, both differences are close to 0. This result is observed for all censoring rates (S8 File).

Even when the meta-analysis is conducted on the logit or the log scale, the extraction error still affects the pooled estimate and its standard error. The patterns of the difference with the correct pooled survival and the difference in standard error are similar to the complementary log-log scale meta-analysis setting (S9 and S10 Files).

## Illustrative example

We conduct meta-analyses of the 10-month progression-free survival in patients with glioma and under systemic therapy. The progression-free survival is obtained from the five studies included in the systematic review by Marwah et al. [8]. Individual data are extracted from the published survival curve of each of the five studies. The sample size in the systemic therapy arm ranges from 17 to 84 patients and the progression-free survival estimates are 0.111, 0.711, 0.093, 0.361 and 0.277. For each study, we then compute the 95%CI using several transformations  f  (S11 File) and extract the standard errors with various transformations  g  and the LRA approach. Table 1 shows a step-by-step example of the use of the LRA approach for one of the studies included in the meta-analysis (study 1). Since the heterogeneity level is important (I^2^ > 80%), a random effects meta-analysis is preferred. Table 2 shows the pooled estimates and 95%CIs.

**Table 1:** Step-by-step example of the implementation of the LRA approach.

For one of the studies included in the meta-analysis (study 1), the survival is 0.111 and its 95%CI computed with the logarithm transformation (i.e., f= ln) is [0.030 to 0.410]. $\hat{S}=\text{0}.\text{1}\text{1}\text{1}$, $LB_{f}=\text{0}.\text{030}$ and $UB_{f}=\text{0}.\text{410 }$ (equation (4))				
**Step 1:** Apply different transformations g to the survival $\hat{S}$ and the bounds $LB_{f}\;$ and $UB_{f}$ of the 95%CI				
g=id g(0.111) = 0.111 g(0.030) = 0.030 g(0.410) = 0.410	g=ln g(0.111) = −2.198 g(0.030) = −3.507 g(0.410) = −0.892	g=ln(−ln) g(0.111) = 0.788 g(0.030) = 1.255 g(0.410) = −0.115	g=logit g(0.111) = −2.081 g(0.030) = −3.476 g(0.410) = −0.364	g=arcsine g(0.111) = 0.340 g(0.030) = 0.174 g(0.410) = 0.695
**Step 2:** Compute the LRA with the different transformationsg				
g = id $\left|\text{ln}\left(\frac{\text{0}.\text{4}\text{1}\text{0}-\text{0}.\text{111 }}{\text{0}.\text{111 }-\text{0}.\text{030 }}\right)\right|$= 1.306	g = ln $\left|\text{ln}\left(\frac{\left(-\text{0}.\text{892})-(-\text{2}.\text{198}\right)}{\left(-\text{2}.\text{198})-(-\text{3}.\text{507}\right)}\right)\right|$= 0.001	g = ln(−ln) $\left|\text{ln}\left(\frac{(-\text{0}.\text{115})-\text{0}.\text{788}}{\text{0}.\text{788}-\text{1}.\text{255}}\right)\right|$= 0.659	g = logit $\left|\text{ln}\left(\frac{\left(-\text{0}.\text{364})-(-\text{2}.\text{081}\right)}{\left(-\text{2}.\text{081})-(-\text{3}.\text{476}\right)}\right)\right|$= 0.207	g = arcsine $\left|\text{ln}\left(\frac{\text{0}.\text{695}-\text{0}.\text{340}}{\text{0}.\text{340}-\text{0}.\text{174}}\right)\right|$ = 0.763
**Step 3:** Identify g yielding the lowest LRA → *ln is yielding the lowest LRA. Thus,* $g^{LRA}\;$ *= ln*				
**Step 4:** Extract the standard error with $g^{LRA}$ → $SE_{\text{ln}\left(\hat{S}\right)}^{extracted}=\frac{\text{ln}(\text{0}.\text{410})-\text{ln}(\text{0}.\text{030})}{\text{2}\cdot \text{1}.\text{9}\text{6}}$ = 0.667				
**Step 5:** Convert $SE_{\text{ln}\left(\hat{S}\right)}^{extracted}$ to the survival scale with the delta method → ${SE}_{\hat{S}}^{extracted}$ = 0.667· 0.111 = 0.074				

**Table 2:** Pooled 10-month progression-free survival (95%CI) obtained by meta-analyses assuming a random effects model.

gf	*id*	*ln*	*ln(-ln)*	*logit*	*arcsine*	LRA^a^
*id*	**0.299** **(0.101 to 0.530)**	0.290 (0.095 to 0.521)	0.288 (0.098 to 0.514)	0.287 (0.096 to 0.513)	0.293 (0.098 to 0.522)	0.2990 (0.101 to 0.530)
*ln*	0.308 (0.106 to 0.538)	**0.299** **(0.101 to 0.530)**	0.292 (0.105 to 0.511)	0.291 (0.103 to 0.512)	0.298 (0.103 to 0.525)	0.299 (0.101 to 0.530)
*ln(-ln)*	0.298 (0.101 to 0.528)	0.297 (0.102 to 0.524)	**0.299** **(0.101 to 0.530)**	0.300 (0.101 to 0.530)	0.298 (0.100 to 0.528)	0.299 (0.101 to 0.530)
*logit*	0.304 (0.103 to 0.535)	0.297 (0.101 to 0.526)	0.300 (0.101 to 0.532)	**0.299** **(0.101 to 0.530)**	0.300 (0.101 to 0.532)	0.299 (0.101 to 0.530)
*arcsine*	0.299 (0.100 to 0.531)	0.304 (0.104 to 0.535)	0.300 (0.102 to 0.529)	0.303 (0.104 to 0.533)	**0.299** **(0.101 to 0.530)**	0.299 (0.101 to 0.530)

The 10-months pooled survival (95%CI) for all combinations of transformations  f  used to compute the 95%CI, and transformation  g  used to extract the standard error from the 95%CI reported rounded to three decimal places.

The meta-analyses are done on the complementary log-log scale using R package *metafor* [7] (version 4.6−0) then the pooled estimate and its 95%CI were converted to the survival scale.

^a^Pooled survival and 95%CI resulting from the use of the transformation  g  designated by LRA for the extraction.

When the correct transformation is applied to extract the standard error, that is  g=f, the pooled estimate (0.299) and its 95%CI are similar across transformations. When g≠f, the pooled estimate ranges from 0.287 to 0.308, that is, an approximately ±3% deviation from the pooled estimate obtained with g=f. The pooled estimate is lower than 0.299 for  f=id and any  g  different from id*.* When  f=ln, a wrong choice of  g  leads to a pooled estimate that is either higher or lower than 0.299, depending on *g*. When f=logit and g=id and when f=arcsine and g=ln, the pooled estimates are greater than 0.299*.* For all other wrong combinations of  f  and g, the deviation from 0.299 is less important, i.e., lower than 1%. The combination f=id and g=logit leads to the most pronounced underestimation of the pooled estimate, i.e., approximately 4%.

The width of the 95%CI was impacted by the choice of a function  g  different from  f  and we generally observe a narrower 95%CI of the pooled survival than in the case where f=g*.* It mainly happens when  f  is the identity or the logarithm transformation but also with the combination of f=ln(−ln) and g=ln*.* Compared to the situations where  g  is equal to f, the most important difference (approximately −5%) in the width of the 95%CI of the pooled survival is obtained with f=ln and g=ln(−ln).

The LRA approach systematically correctly identifies the transformation  f  and the pooled estimates are equal to those obtained with the correct combination of f and g.

## Discussion

This study shows that extraction errors caused by an incorrect assumption about the transformation used to compute the 95%CI of the survival mainly arise when the survival is close to 0 or 1, or the effective sample size is small. The magnitude and frequency of the errors decrease as the number of decimal places used for reporting the survival and 95%CI increases. Rounding the 95%CI bounds introduces a non-monotonic error in the extracted standard error as the rounded interval may be wider or narrower than the unrounded one depending on the sample size. The magnitude and frequency of the extraction error also depend on the pair of transformations  f  used to compute the 95%CI and  g  used to extract the standard error. These extraction errors eventually carry over into errors in the results of the meta-analysis through the weights (inverse of variance) attributed to each study. The standard error of the pooled survival estimate is more sensitive to the extraction errors than the pooled estimate itself as the former depends solely on the value of the extracted variance. This is even more true for fixed effects than random effects meta-analyses since in the first case the extracted variance is the extracted standard error squared whereas in the second case, the between-study variance is added to the extracted variance and partly offsets the extraction error. Thus, the higher the between study variability is, the lower should be the extraction error impact on the weights attributed to each study included in the meta-analysis and thereby on the standard error of the pooled estimate.

To overcome this issue, we propose to use the asymmetry of the 95%CI of the survival to correctly identify the transformation used to compute that 95%CI and thereby enhance the extraction of the standard error. Overall, this easily applicable approach, called the LRA approach, exhibits a satisfactory performance in identifying the said transformation and the effects are notable on the meta-analysis results as it yields pooled estimates and standard errors similar to those obtained in the absence of extraction error. Cases where the LRA approach tends to fail in the identification of the computation function are when the sample size is large or the survival is around 0.20, 0.40 or 0.50, or when it is close to 0 or 1 or when the 95%CI of the survival is truncated. The LRA approach tends to fail to distinguish between the 95%CIs computed with the identity, logit and arcsine transformations for values of the survival around 0.5. The 95%CI of survival obtained with the identity transformation is always symmetric. The 95%CIs of survival around 0.5 computed with the arcsine and logit transformations also become symmetric because both functions  f  are centrally symmetric around the point (0.5, *f*(0.5)). The 95%CI of a survival around 0.4 computed with the complementary log-log transformation is symmetric because the function becomes quasi-linear in this region (specifically around *1/e*). Regarding the 95%CI of the survival close to 1 computed with the logarithm transformation, it is symmetric because the logarithm function is approximately linear near 1. When the survival is close to 0, the LRA approach fails to distinguish between the 95%CIs computed with logit and logarithm because both transformations behave similarly in that region. For survival around 0.2, the 95%CIs computed with arcsine and complementary log-log transformations are similar due to similarities in the asymmetry created by these functions and their equality at 0.2 which makes the LRA approach unable to distinguish between them (S1 File). Asymptotically, all transformations produce the same 95%CI which explains why the range of values for which the LRA approach fails to identify the correct transformation increases as the sample size increases.

We created a Shiny App for the implementation of this approach (https://vvwdt0-marilyn-ello.shinyapps.io/LRA_approach_app/). When given the survival and its associated 95%CI, the Shiny App returns the transformation designated as the transformation used to compute the 95%CI and the extracted standard error on five different scales.

This study presents some limitations. Although other methods can be used to compute the 95%CI of a survival, we focused on those using transformation of the estimate and the LRA approach is valid for the set of transformations considered. There exist non-transformation-based methods such as bootstrap procedures [9] or likelihood-based ones [10] but the LRA approach assumes that confidence intervals are constructed from the standard error. If the standard error is obtained by bootstrap, the 95%CI could have been computed using a transformation and thus be based on the standard error. Therefore, the LRA approach remains applicable. However, the LRA approach cannot detect a situation where the percentile method is used as the symmetry of the interval depends on the data and not on the computation method (S4 File). Likelihood-based 95%CIs will not be identified by the LRA approach either, since they are not standard error-based. If the LRA approach is applied to a 95%CI that is not based on a standard error, the extracted standard error might be inaccurate but will be the most consistent with the structure of the 95%CI. Also, we assumed exponential distributions for survival and censoring times to compute extraction error and in the simulation study. Nevertheless, the underlying censoring and survival times distributions only have an impact on the extraction and the LRA approach results through the effective sample size (S3 File). For a given survival, if two distributions of censoring times yield the same effective sample size—thus the same 95%CI—, the LRA approach yields the same result and the extracted standard errors are equal. If the two distributions of censoring times yield different effective sample sizes, the LRA approach yields two different extracted standard errors. In other words, varying the distribution of censoring times is equivalent to varying the effective sample size. Regarding the distribution of survival times, the time at which the survival of interest is obtained is not determinant for the extraction of the standard error, only the values of the survival and 95%CI are. If there is no censoring, a given survival obtained from a study of sample size *n* will always have the same 95%CI whatever the underlying distribution of survival times. If there is censoring, the reasoning about the distribution of censoring times applies. Thus, the cases studied cover the relevant spectrum of cases that can be encountered that is, a given survival and different effective sample sizes or different 95%CIs. Despite the limitations, the results regarding the performance of the LRA are strongly reliable. Even if the impact of the extraction can be modest, rather than extracting the standard error using an arbitrarily chosen transformation, the LRA approach provides a structured alternative with reliable performance.

## Supporting information

S1 TableFunctions used to compute 95%CIs of the survival $\hat{S}$ and expression of standard errors $SE_{f\left(\hat{S}\right)}$ obtained with the delta method.(PDF)

S1 File Theoretical justification of the LRA approach. (PDF)

S2 File Decision rule of LRA approach for truncated 95%CIs. (PDF)

S3 File Survival and censoring times distributions and effective sample size. (PDF)

S4 File Extraction error and LRA approach performance for bootstrap-generated 95%CIs. (PDF)

S5 File Data generation procedures for the simulation studies. (PDF)

S6 File Extraction error. (PDF)

S7 File LRA approach performance and resulting extraction error. (PDF)

S8 File Impact of extraction error and LRA approach on meta-analysis on the complementary log-log scale. (PDF)

S9 File Impact of extraction error and LRA approach on meta-analysis on the logit scale. (PDF)

S10 File Impact of extraction error and LRA approach on meta-analysis on the log scale. (PDF)

S11 File 95%CIs of studies included in the meta-analyses of the illustrative example. (PDF)
