# Supplementary material for: Extracting standard error from the 95% confidence interval of a survival
Source: PLoS One. 2026 Aug 4;21(8):e0355291. doi: 10.1371/journal.pone.0355291 (PMC13436750; doi:10.1371/journal.pone.0355291)
Supplement: Supplementary file 1 [file pone.0355291.s001.pdf]

S1 Table. Functions used to compute 95%CIs of the survival $\widehat{S}$ and expression of standard errors $SE_{f(\widehat{S})}$ obtained with the delta method.

$f(.)$	$f^{-1}(.)$	$SE_{f(\hat{S})}$
Identity<br>*id(.)*	Identity<br>*id(.)*	$SE_{\hat{S}} = \hat{S} \cdot SE_{\ln(\hat{S})}$
Logarithm<br>*ln(.)*	Exponential<br>*exp(.)*	$SE_{\ln(\hat{S})} = \frac{SE_{\hat{S}}}{\hat{S}}$
Complementary log-log<br>*ln(-ln(.))*	*exp(-exp(.))*	$SE_{\ln\left(-\ln(\hat{S})\right)} = \frac{-SE_{\hat{S}}}{\hat{S}\ln\left(\hat{S}\right)}$
Logit<br>*logit(.)*	*1/(1+exp(-.))*	$SE_{\text{logit}(\hat{S})} = \frac{SE_{\hat{S}}}{\hat{S}(1-\hat{S})}$
Arcsine<br>*sin⁻¹* ($\sqrt{}$)	*sin²(.)*	$SE_{arcsine(\sqrt{\hat{S}})} = \frac{SE_{\hat{S}}}{2\sqrt{\hat{S}(1-\hat{S})}}$
