# Supplementary material for: Extracting standard error from the 95% confidence interval of a survival
Source: PLoS One. 2026 Aug 4;21(8):e0355291. doi: 10.1371/journal.pone.0355291 (PMC13436750; doi:10.1371/journal.pone.0355291)
Supplement: Supplementary file 2 [file pone.0355291.s002.pdf]

Theoretical justification of the LRA approach

This document presents the theoretical justification of the LRA approach. The 95%CI of a survival can be computed using different transformations. These transformations produce different 95%CI of the survival especially in terms of asymmetry. The LRA approach exploits the information about the asymmetry to identify the transformation that was used to compute the said interval. The identified function is then used to extract the standard error.

95%CI and asymmetry

To compute the 95%CI of a survival $\hat{S}$ using a transformation f, we assume that $f(\hat{S})$ is normally distributed and first compute its 95%CI:

$$[f(\hat{S}) - z\ SE_f\ ;\ f(\hat{S}) + z\ SE_f]$$

With z the 0.975-quantile of the standard normal distribution and SE_f the standard error of $f(\hat{\mathrm{S}})$ obtained with the delta method. This 95%CI is symmetric. Then, the 95%CI of $\hat{\mathrm{S}}$ is obtained by back transforming the bounds of the 95%CI of $f(\hat{\mathrm{S}})$ with f^{-1}, the inverse of the transformation f. The lower and upper bounds of the 95%CI of $\hat{S}$, denoted L and U respectively, are given by[1]:

[1]If f is decreasing, the bounds are reversed.

$$L = f^{-1}(f(\hat{S}) - k) \text{ and } U = f^{-1}(f(\hat{S}) + k) \text{with } k = z\ SE_f$$

If the 95%CI of $\hat{S}$ is symmetric, $U - \hat{S} = \hat{S} - L$ which is equivalent to $\ln\left(\frac{U-\hat{S}}{\hat{S}-L}\right) = 0$.

$\ln\left(\frac{U-\hat{S}}{\hat{S}-L}\right)$ denoted κ_f is an index of the asymmetry level of the 95%CI of $\hat{S}$ computed with the transformation f. The more κ_f deviates from 0, the more asymmetric the 95%CI is.

Characterization of the asymmetry level

For readability purposes, we will refer to the inverse of f as l. The second order[2] Taylor expansions of L and U are:

$$L \approx l(f(\hat{S})) - l'(f(\hat{S}))\ k + \frac{1}{2}l''(f(\hat{S}))k^2 U \approx l(f(\hat{S})) + l'(f(\hat{S}))\ k + \frac{1}{2}l''(f(\hat{S}))k^2$$

with $l(f(\hat{S})) = \hat{S}$, we obtain:

$$\hat{S} - L \approx l'(f(\hat{S}))\ k - \frac{1}{2}l''(f(\hat{S}))k^2 U - \hat{S} \approx l'(f(\hat{S}))\ k + \frac{1}{2}l''(f(\hat{S}))k^2$$

Thus, we have:

$$\kappa_f = \ln\left(\frac{U-\hat{S}}{\hat{S}-L}\right) \approx \ln\left(\frac{l'(f(\hat{S}))\ k + \frac{1}{2}l''(f(\hat{S}))\ k^2}{l'(f(\hat{S}))\ k - \frac{1}{2}l''(f(\hat{S}))\ k^2}\right) = \ln\left(\frac{1 + \frac{1}{2}\frac{l''(f(\hat{S}))}{l'(f(\hat{S}))}k}{1 - \frac{1}{2}\frac{l''(f(\hat{S}))}{l'(f(\hat{S}))}k}\right)$$
$$= \ln\left(1 + \frac{1}{2}\frac{l''(f(\hat{S}))}{l'(f(\hat{S}))}k\right) - \ln\left(1 - \frac{1}{2}\frac{l''(f(\hat{S}))}{l'(f(\hat{S}))}k\right)$$

[2]Higher order terms are negligible.

by Taylor expansion of $\ln(1+x)$, we obtain:

$$\kappa_f \approx \frac{1}{2}\frac{l''(f(\hat{S}))}{l'(f(\hat{S}))}k + \frac{1}{2}\frac{l''(f(\hat{S}))}{l'(f(\hat{S}))}k = \frac{l''(f(\hat{S}))}{l'(f(\hat{S}))}k$$

By the inverse of function theorem, for $f'(x) \neq 0$, we have:

$$\begin{aligned}\kappa_f \approx \frac{l''(f(\hat{S}))}{l'(f(\hat{S}))}k &= \frac{-f''(\hat{S})/(f'(\hat{S}))^3}{1/f'(\hat{S})}k \\ &= -\frac{f''(\hat{S})}{f'(\hat{S})^2}k\end{aligned}$$

Knowing that $SE_f = f'(\hat{S})\ SE$ (delta method) with SE the standard error of $\hat{S}$, $k = z\ f'(\hat{S})\ SE$.

Finally, we obtain:

$$\kappa_f = \ln\left(\frac{U-\hat{S}}{\hat{S}-L}\right) \approx -\frac{f''(\hat{S})}{f'(\hat{S})^2} z f'(\hat{S}) SE = -\frac{f''(\hat{S})}{f'(\hat{S})} z\ SE$$

Thus, the level of asymmetry of the 95%CI of $\hat{S}$ computed with the transformation f is $\kappa_f \approx -\frac{f''(\hat{S})}{f'(\hat{S})} z\ SE$ which depends on the transformation, the value of the survival and the effective sample size n' because $SE = \sqrt{\frac{\hat{S}(1-\hat{S})}{n'}}$. A given 95%CI is symmetric if $f''(\hat{S}) = 0$.

Difference in asymmetry level

Two transformations f_1 and f_2 lead to two 95%CIs with the same asymmetry level if $\kappa_{f_1} - \kappa_{f_2} = 0$. Table 1 presents the difference in asymmetry between these two 95%CIs f and the values of $\hat{S} \in]0;1[$ for which they have the same asymmetry level.

Table 1. Difference in asymmetry between two 95%CIs computed with two different f and values of $\hat{S} \in]0;1[$ for which they have the same asymmetry level.

**Pair** $(f_1;\ f_2)$	$\kappa_{f_1} - \kappa_{f_2}$	root of $\kappa_{f_1} - \kappa_{f_2}$ in $]0;1[$
identity − log	$1/\hat{S}$	$\emptyset$
identity − logit	$-\frac{2\hat{S}-1}{\hat{S}(1-\hat{S})}$	$\hat{S} = 0.5$
identity − arcsine	$-\frac{2\hat{S}-1}{2\hat{S}(1-\hat{S})}$	$\hat{S} = 0.5$
identity − comp. log-log	$\frac{1+\ln(\hat{S})}{\hat{S}\ln(\hat{S})}$	$\hat{S} = 1/e \approx 0.37$
log− logit	$-\frac{1}{1-\hat{S}}$	$\emptyset$
log− arcsine	$-\frac{1}{2\hat{S}(1-\hat{S})}$	$\emptyset$
log− comp. log-log	$\frac{1}{\hat{S}\ln(\hat{S})}$	$\emptyset$
logit − arcsine	$\frac{2\hat{S}-1}{2\hat{S}(1-\hat{S})}$	$\hat{S} = 0.5$
logit − comp. log-log	$\frac{\hat{S}\ln(\hat{S})+1-\hat{S}}{\hat{S}\ln(\hat{S})(1-\hat{S})}$	$\emptyset$
arcsine − comp. log-log	$\frac{-2\hat{S}+\ln(\hat{S})+2}{2\hat{S}\ln(\hat{S})(1-\hat{S})}$	$\hat{S} \approx 0.2$ (no closed-form solution)

Two transformations yield the same asymmetry when $\hat{S}$ is around 0.20 (complementary log-log & arcsine), $1/e$ (identity & complementary log-log) or 0.50 (identity, logit & arcsine). For almost every survival values, the asymmetry of the back transformed 95%CI is a near-unique signature of the transformation that was used to compute it. Since the asymmetry of that back-transformed interval is determined by the second derivative of the generating transformation at $\hat{S}$, finding the transformation that removes this asymmetry — that is, the transformation under which the bounds become symmetric — is equivalent to identifying the transformation that originally produced it. This is precisely the property exploited by the LRA approach. This approach identifies the transformation used to compute the 95%CI of $\hat{S}$ by selecting the transformation which, when applied to the bounds, yields the most symmetric transformed 95%CI. The transformation identified is then used to extract the standard error.

The ambiguous cases

Ambiguous cases are the cases where two transformations lead to 95%CIs with similar asymmetry levels. The asymmetry levels created by different transformations coincide only at the three values of survival identified in the previous section. Also, When $n' \to \infty$, $\kappa_f \to 0$ and all 95%CIs are symmetric. Also, as discussed in the manuscript, the LRA approach fails to identify the 95%CI computed with the logarithm transformation, because this transformation is approximately linear near 1 hence behaves like the identity transformation. When the survival is close to 0, the LRA approach fails to distinguish between the 95%CIs computed with logit and logarithm because both transformations behave similarly in that region.

Two complementary signals make ambiguous cases easy to recognise, with no additional computation. First, the analysis above identifies in advance the observed survivals at which two transformations can have the same asymmetry: $\hat{S} \approx 0.20$, $\hat{S} \approx 0.37$ and $\hat{S} = 0.50$ and values of survival close to 0 or 1. A reported survival near one of these values calls for caution as well as cases where the effective sample size is high. Second, the LRA approach comes with its own diagnostic. For each interval this approach computes the logarithm of the relative asymmetry (LRA) of the 95%CI transformed with every candidate transformation thus the analyst only has to inspect it: the selected transformation is the one retuning the smallest LRA. If the two smallest LRAs are close, the transformed 95%CI is (almost) symmetric on two scales. The gap between the smallest and the next-smallest LRA is therefore an immediately available indicator: a clear gap means unambiguous identification, a small gap suggests ambiguity. In conclusion, ambiguous cases are both predictable and identifiable.
