# Supplementary material for: Extracting standard error from the 95% confidence interval of a survival
Source: PLoS One. 2026 Aug 4;21(8):e0355291. doi: 10.1371/journal.pone.0355291 (PMC13436750; doi:10.1371/journal.pone.0355291)
Supplement: Supplementary file 3 [file pone.0355291.s003.pdf]

LRA approach extended to truncated cases

In the cases where the 95%CI is truncated and the LRA approach returns no compatible transformation that can lead to truncated bounds, we suggested to we chose g^{LRA} = *id* if the lower bound is truncated and g^{LRA}=*ln* if the upper bound is truncated. This file presents the rationale behind this decision rule. For readability purposes, we refer to upper bounds equal to 1 and lower bounds equal to 0 as truncated either due actual truncation or rounding.

We generated effective sample sizes *n'* between 10 and 1000 by step of 5. For each *n'*, we computed all survival estimates associated with a number of survivors $s \in [1; n'-1]$. For each survival, the standard error was obtained by $\sqrt{\frac{\hat{S}(1-\hat{S})}{n'}}$. We computed five 95%CIs, each corresponding to the use of one transformation within the set of identity, logarithm, complementary log-log, logit and arcsine. From each 95%CI, we apply the LRA approach to retrieve the number of cases leading to no compatibility. We focused on the few cases where either the upper or the lower bound is truncated. The percentages of cases where one bound is truncated among all the cases considered is shown in Table 1. This percentages remain very low. Table 2 presents for each transformation and number of decimal places, the percentage of cases leading to no compatibility among the cases of truncated bounds.

The identity transformation, more than for any other transformation, leads to cases where LRA approach yields no compatibility when the lower bound is truncated. The same logic applies to the logarithm when the upper is truncated.

Whether a transformation is compatible with a given 95%CI is deterministic functions $\hat{S}$ and the 95%CI bounds, thus the effective sample size. For given survival and effective sample size, the rounding of the reported 95%CI, the set of compatible transformations returned by the LRA approach are determined without randomness. Thus, our approach does not rely on Monte-Carlo simulations but sweeps the pairs of survival and effective sample size.

Table 1. Percentages of cases where a bound is truncated among all the cases considered.

Transformation	Truncated bound	4 decimal places	3 decimal places	2 decimal places
Identity	Upper	0.6	0.7	1.5
	Lower	0.6	0.7	1.5
Logarithm	Upper	0.6	0.8	1.5
	Lower	0	0.1	1.0
Complementary log-log	Upper	0	0.1	1.0
	Lower	0	0.2	1.1
Logit	Upper	0	0.1	1.0
	Lower	0	0.1	1.0
Arcsine	Upper	0.2	0.3	1.2
	Lower	0.2	0.3	1.2

Table 2. Percentages of cases where the LRA approach returns no compatible transformation.

Transformation	Truncated bound	4 decimal places	3 decimal places	2 decimal places
Identity	Upper	0	2.1	11.4
	Lower	0	4.1	12.3
Logarithm	Upper	0	4.0	12.4
	Lower	(*)	0	0
Complementary log-log	Upper	(*)	0	0
	Lower	(*)	0	0.1
Logit	Upper	(*)	0	0
	Lower	(*)	0	0
Arcsine	Upper	0	0	2.1
	Lower	0	0	2.3

(*) Not truncated
