# Supplementary material for: Extracting standard error from the 95% confidence interval of a survival
Source: PLoS One. 2026 Aug 4;21(8):e0355291. doi: 10.1371/journal.pone.0355291 (PMC13436750; doi:10.1371/journal.pone.0355291)
Supplement: Supplementary file 4 [file pone.0355291.s004.pdf]

Survival and censoring distributions and effective sample size

The effective sample size is, by definition, the sample size that an uncensored analysis would require to estimate the same standard error for a given survival as an analysis with censoring. The effective sample size is obtained by equating the standard error *SE* of a survival $\hat{S}$ to $\sqrt{\frac{\hat{S}(1-\hat{S})}{n'}}$, with *n′* the effective sample size. In the case of an uncensored survival analysis, *n′* is equal to the sample size. Whatever the underlying survival and censoring times distributions, the 95%CI of a given survival $\hat{S}$ is a deterministic function of $\hat{S}$ and its associated standard error *SE*. Thereby, the 95%CI of $\hat{S}$ can be expressed as a function of $\hat{S}$ and *n′*. If two studies with two different underlying censoring and survival times distributions report the same survival and 95%CI, it means that they have the same *n′*. If the two studies report different 95%CIs for a given survival, it means that they have different *n*'. Thus, the effective sample size alone captures the underlying distribution of survival and censoring times. As for the extraction error, it arises from a difference between the transformation used to compute the 95%CI of the survival and the transformation used to extract the standard error from that interval. Therefore, the results of our study are not specific to one underlying distribution of survival or censoring times but any combination of the two distributions that produces a given survival $\hat{S}$ and associated 95%CI (or effective sample size).

1. Relationship between standard error and effective sample size

To show that the standard error —thus its extraction— depends only on the survival and effective sample size, we assessed empirically the relationship between the sample size, the effective sample size and the standard error of given survival and censoring rate for five combinations of survival and censoring times distributions.

1.1 Calibration of distributions

Survival and censoring times were generated from either exponential or Weibull distributions whose parameters were calibrated to yield a target survival *S* and censoring rate *r* at time *t*.

For survival times, when an exponential distribution was assumed, the hazard λ_{surv} was derived with the closed form solution: $\lambda_{surv} = -\frac{\ln(S)}{t}$. When a Weibull distribution was assumed, the shape parameter *k* was fixed and the scale parameter σ was obtained as $\sigma = \frac{t}{(-\ln(S))^{\frac{1}{k}}}$.

The generation of censoring times takes into account the survival times and the target censoring rate. Since there is no close form solution, the parameters of both the exponential and Weibull distributions were found numerically solving the empirical probability of being censored before the time *t* being equal to a target censoring rate. To do so, we first generated 200000 individual survival times from the calibrated survival times distribution (either exponential or Weibull distribution depending on the case considered). For exponential censoring times distribution, 200000 censoring times were generated

from an exponential distribution with candidate hazard λ_{cens} between 10^{-6} and 50. The λ_{cens} minimizing the difference between the target censoring rate and the empirical censoring probability (proportion of individual being censored before time *t*) was found using Brent's root-finding algorithm (function *uniroot* in R). The same procedure was applied for Weibull censoring times for *k* being fixed and the scale parameter σ obtained by numerical optimization with candidate between 0.01 and 100.

1.2 Simulations scenarios

Once the parameters of the distributions were determined, the survival and censoring times were generated and the Kaplan-Meier survival analysis was performed using the package *survival*. The survival and its standard error at the time of interest were obtained and the corresponding effective sample size computed.

This process was repeated for target survival of 0.1, 0.25, 0.5, 0.75 and 0.9, for target censoring rate equal to 50%, sample sizes equal to 50, 100, 300, 700 and 1000 and five combinations of survival and censoring time distributions (survival/censoring) and fixed shape parameters *k*:

- exponential/exponential
- Weibull (*k* = 1.5)/exponential
- exponential/Weibull (*k* = 0.7)
- two Weibull/Weibull combinations:
    - late events and early censoring: Weibull (*k*=1.5)/Weibull (*k* = 0.7); referred to as weibull/weibull (+)
    - early events and late censoring: Weibull (*k*=0.7)/Weibull (*k* = 1.5); referred to as weibull/weibull (-)

1.3 Results

Figure 1 presents the relationship between the standard error and sample size (left panels), and the relationship between the standard error and the effective sample size (right panels) for a given survival.

When the standard error is plotted against the sample size, the combinations of distributions are distinguishable especial for survival close to 0 or 1. This means that a given survival estimated from a study with a given sample size will have different associated standard errors depending on the underlying combination of survival and censoring times distributions.

When the standard error is plotted against the effective sample size, the combinations of distributions collapse into a single curve, except for the Weibull/Weibull(-) when the survival is small (0.1) and the censoring rate is high (50%) which is an artifact. This means that a given survival estimated from a study with a given effective sample size will have the same associated standard error regardless of the underlying combination of survival and censoring times distributions. Therefore, when the effective sample size is known, the information about the underlying distributions become irrelevant when showcasing the extraction error.

Fig 1. Relationships between sample size, effective sample size and standard error

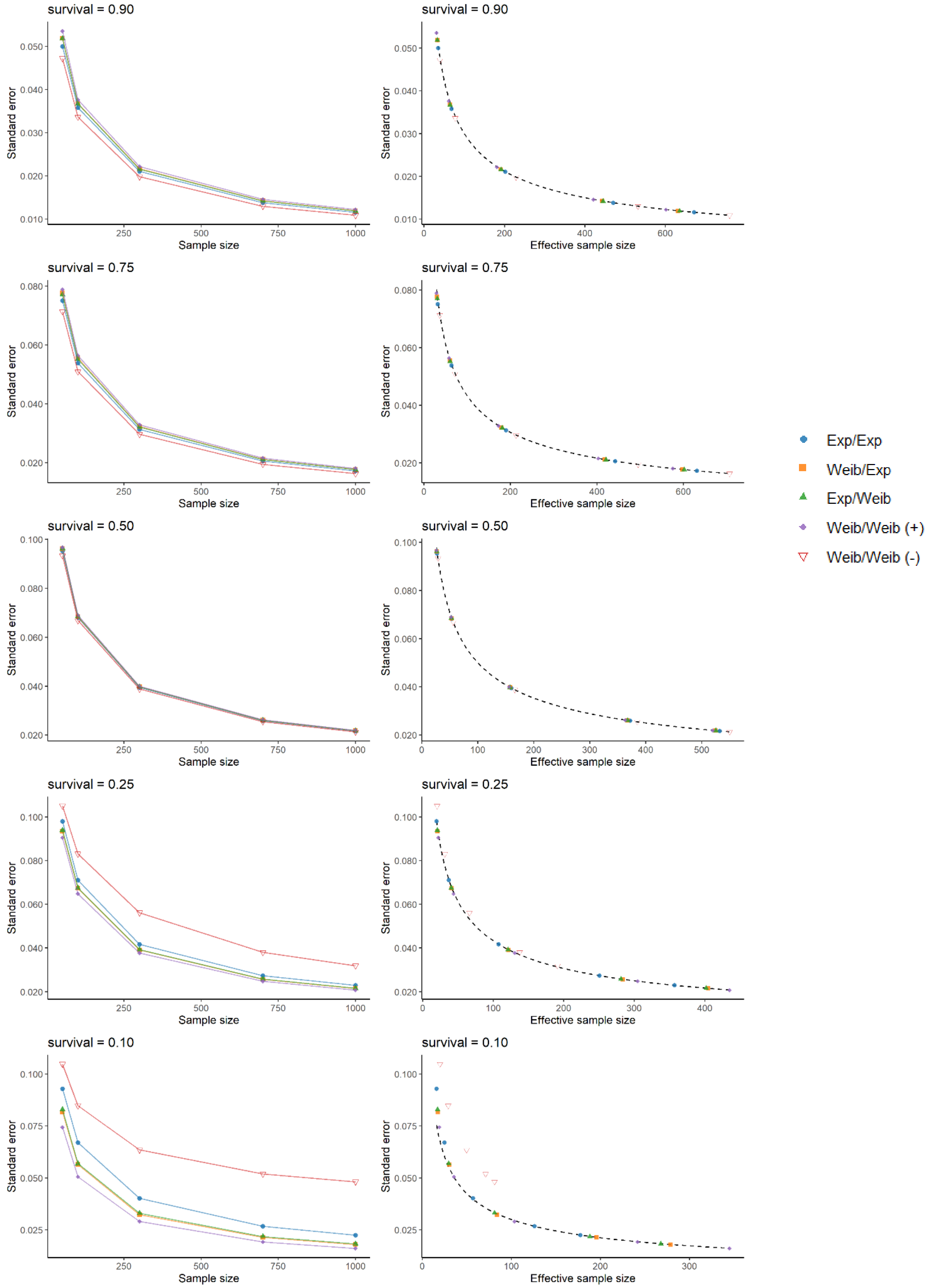

2. Extraction error and LRA approach performance plots

To make our analysis more comprehensive, we present the extraction error and performance of the LRA approach in the manuscript as a function of the effective sample size. To help the readers' interpretation of the results, Table 1 shows the effective sample size expressed as a percentage of the total sample size across different scenarios. The values can be interpreted as follows: for a given scenario and survival probability, the effective sample size corresponds to *x*% of the total sample size. The table allows a reader to determine the expected extraction error or LRA performance for a study of a given survival, censoring rate, and underlying censoring and survival distributions shape.

Table 1 was obtained as follows:

1. The same combinations of survival and censoring times as in Section 1 were used and they were calibrated the same way.

2. Once the parameters of the distributions were determined, the survival and censoring times were generated for 100000 individuals and the Kaplan-Meier survival analysis was performed using the package *survival*. The survival and its standard error at the time of interest were obtained and the corresponding effective sample size computed.

3. The effective survival was then divided by 100000

4. Steps 1-3 were repeated for survival between 0.1 and 0.9 by step of 0.1 and censoring rate of 25% and 50%.

Table 1. Effective sample size as percentage of sample for different censoring rates and combinations of survival/censoring times distributions

Survival	Censoring (%)	Exp/Exp	Weib/Exp	Exp/Weib	Weib/Weib (+)	Weib/Weib (−)
**0.9**	25	85.2	82.9	83.2	81.5	89.7
	50	66.9	63.2	63.5	60.2	75.9
**0.8**	25	84.1	82.0	82.3	80.5	88.6
	50	64.5	60.8	61.2	58.5	72.2
**0.7**	25	82.5	80.8	81.0	79.7	87.1
	50	61.4	58.2	58.8	56.5	68.3
**0.6**	25	80.9	79.6	79.5	78.0	85.0
	50	57.8	55.7	55.8	54.6	62.6
**0.5**	25	79.0	77.6	77.9	77.0	82.2
	50	52.9	52.2	52.4	51.7	54.9
**0.4**	25	76.3	75.7	75.8	75.4	78.2
	50	47.2	48.0	48.4	49.1	43.3
**0.3**	25	72.1	72.9	72.6	73.5	72.0
	50	40.1	43.5	42.9	45.6	28.0
**0.2**	25	67.0	68.9	68.6	70.5	61.1
	50	30.6	37.2	37.0	41.0	8.2
**0.1**	25	56.2	62.5	61.9	65.8	38.2
	50	17.4	28.0	26.5	34.8	0.2

3. Meta-analyses

The results of meta-analyses depend on the data from the studies included in it. The only study data used for the meta-analysis of survival are the survival and the standard error. Since each standard error is itself determined by the survival and effective sample size alone, the entire pooled analysis depends on the event and censoring distributions only through the per-study effective sample sizes. Two meta-analyses whose studies match, study by study, on survival and effective sample size yield identical pooled estimates whatever the underlying censoring and survival times distributions of the studies included in each meta-analysis.

To assess this empirically, we simulated meta-analyses under each of the five combinations of distributions of Section 1. The meta-analysis plan and output generation follow the ones from the main analysis in the manuscript. The only difference is that here, the sample size considered are 30, 100 and 300.

Table 2 summarizes for each combination of underlying distribution the minimum and maximum difference with the correct pooled estimate and difference in SE across all sample sizes, survival, method of computation of the 95%CI of the survival and method of extraction of the standard error. The table shows that the impact of the method of extraction of the standard error on the meta-analysis results is similar across the assessed distributions of survival and censoring times.

Table 2. Difference with the correct pooled survival and difference in standard error for all combinations of survival and censoring times distributions

	Exp/Exp	Weib/Exp	Exp/Weib	Weib/Weib (+)	Weib/Weib (-)
**Fixed effects**					
Diff. in SE, max (%)	25.1	23.3	26.5	23.2	25
Diff. in SE, min (%)	-5	-5	-5	-4.9	-5.1
Diff. with correct pooled survival, max	0.01	0.01	0.01	0.01	0.01
Diff. with correct pooled survival, min	-0.03	-0.02	-0.03	-0.02	-0.0.3
**Random effects**					
Diff. in SE, max (%)	13	13.2	13.3	13.5	12.9
Diff. in SE, min (%)	-3.4	-3.4	-3.5	-3.5	-3.2
Diff. with correct pooled survival, max	0.02	0.02	0.02	0.02	0.02
Diff. with correct pooled survival, min	-0.03	-0.03	-0.03	-0.03	-0.02
