# Supplementary material for: Extracting standard error from the 95% confidence interval of a survival
Source: PLoS One. 2026 Aug 4;21(8):e0355291. doi: 10.1371/journal.pone.0355291 (PMC13436750; doi:10.1371/journal.pone.0355291)
Supplement: Supplementary file 5 [file pone.0355291.s005.pdf]

Performance of the LRA approach on 95%CI obtained with bootstrap procedure

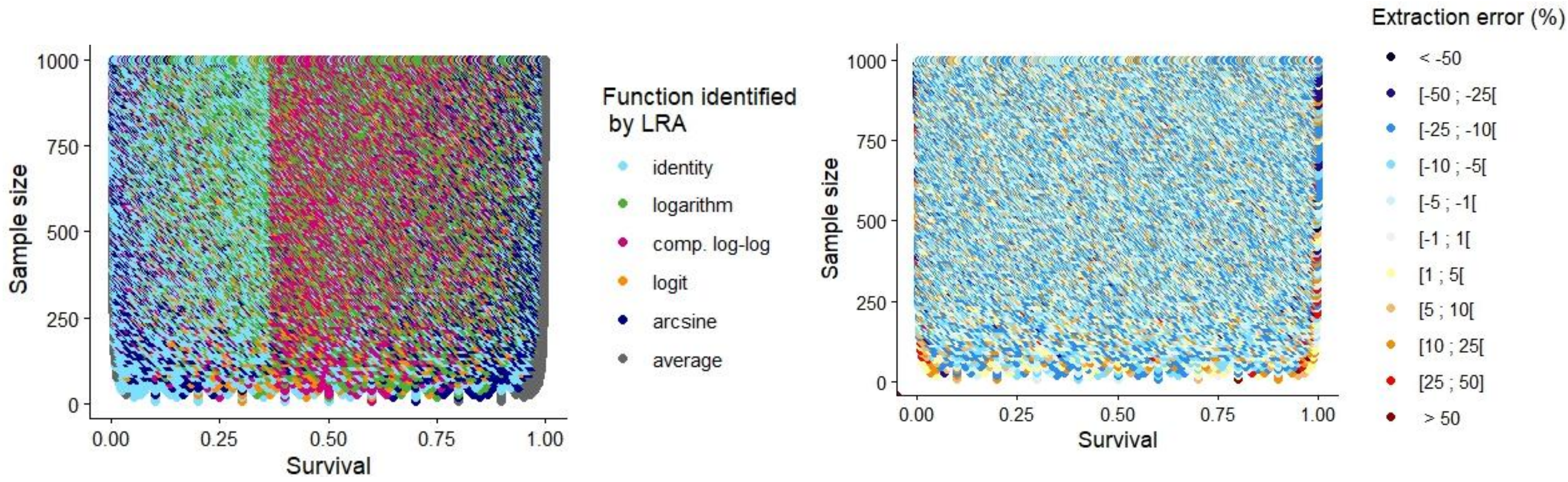

Fig 1. Results of the application of the LRA approach on 95%CI of survival computed with bootstrap percentile and reported round to 3 decimal places.
Left panel: function suggested by the LRA approach for the extraction of the standard error from the 95%CI;
Right panel: Extraction error resulting from the use of the function suggested by the LRA approach for the extraction of the standard error.

We considered sample sizes ranging from 10 to 1000 in increments of 10. For a given sample size *n*, all possible numbers of survivors *s* from 1 to *n*-1 were considered. Assuming no censoring, the survival parameter is defined as $S = \frac{s}{n}$. For each combination of sample size and value of survival parameter, a sample was generated from a binomial distribution. Then, 5000 bootstrap replicates were drawn from each generated sample. For each bootstrap replicate, the survival was computed as $\hat{S} = \frac{\hat{s}}{n}$, with $\hat{s}$ the number of survivors in the bootstrap sample. The 95%CI of the survival associated with a given *n* and *S* was obtained with the bootstrap percentile method over the 5000 replicates. The LRA approach was then applied on the bootstrap-derived 95%CI rounded to three decimal places for all combinations of *s* and *n*.

The left panel of Fig 1 shows the functions returned by LRA for all combinations of *s* and *n* considered. The right panel of the same figure shows the extraction error. This error was computed as the difference in percents between the standard error extracted after applying the LRA approach and the one computed with the binomial approximation, i.e. $\sqrt{\frac{S(1-S)}{n}}$.

The LRA approach does not identifies when a 95%CI is obtained via bootstrap; it returns the function leading to the smallest asymmetry of the transformed 95%CI. In most cases, the extraction error falls between -5% and 5%. The extraction error becomes more important for survival close to 1 or for small sample sizes.
