# Supplementary material for: Extracting standard error from the 95% confidence interval of a survival
Source: PLoS One. 2026 Aug 4;21(8):e0355291. doi: 10.1371/journal.pone.0355291 (PMC13436750; doi:10.1371/journal.pone.0355291)
Supplement: Supplementary file 6 [file pone.0355291.s006.pdf]

Data generation procedures for simulation studies

Fixed-effects meta-analyses

Each simulated meta-analysis includes ten studies of the same sample size (either 30, 50, 100, 150 or 300). For each study, survival times are randomly generated from an exponential distribution with a hazard λ_{surv} equal to 0.05. Censoring times are also randomly generated from an exponential distribution but with a hazard λ^{*}_{cens} computed to correspond to a given level of censoring (either 0%, 25% or 50%) common to all studies included in the meta-analysis. The hazard of censoring times is computed as described by Nemes et al. [1]:

$$\lambda^{*}_{cens} = argmin_{\lambda_{cens}} \left[\left(\frac{\lambda_{cens}}{\lambda_{surv} + \lambda_{cens}}\right)\left(1 - e^{-(\lambda_{surv}+\lambda_{cens})t}\right) - censoring\ level\right] \qquad (1)$$

Where $censoring\ level$ is the desired censoring rate (0, 25% or 50%) and t is the timepoint corresponding to the survival of interest. Since the survival times are generated from an exponential model, the theoretical survival probability at time t is given by $S(t) = e^{-\lambda_{surv}t}$. Accordingly, the time point t corresponding to a specified survival probability of interest is $t = -\frac{\ln(S(t))}{\lambda_{surv}}$.

Random-effects meta-analyses

In the random effects model settings, the data are generated in similar way to the fixed effects model ones. The difference resides in the generation of survival times. In this case, hazard used for the generation of the survival times of studies included in the same meta-analysis differ from one another to introduce heterogeneity. For a given meta-analysis, ten values — since ten studies are included in one meta-analysis — of hazard are randomly generated from a log-normal distribution. To do so, we first randomly generate ten values according to a normal distribution with mean *ln*(0.05) and standard deviation equal to 0.8. Then, generated values are transformed with the exponential function. The ten transformed values are the hazards used to generate the survival times for each of the ten studies included in the meta-analysis.

Continuity correction

A continuity correction is applied to survival estimates equal to 0 or 1 leading to survival of $\frac{0.5}{n+1}$ and $\frac{n+0.5}{n+1}$ respectively with n being the sample size.

To obtain the standard error of a given continuity corrected survival in order to include it in a meta-analysis, we:

1) compute the average effective sample size of the studies included in the same meta-analysis whose survival are not continuity corrected
2) use the compute average effective sample size to compute stand as follows:

$$\sqrt{\frac{\widehat{S}'(1-\widehat{S}')}{\bar{n}'}} \qquad (2)$$

With $\widehat{S}'$ the continuity corrected survival and $\bar{n}'$ the average effective sample size.

To include a study whose survival is continuity corrected in a meta-analysis, we use the standard error computed as in equation (2) to obtain the weights. Meta-analysis including at least one study whose survival is continuity corrected are included in the assessment of the impact of the extraction errors but not in the assessment of the LRA approach on the meta-analysis results.

Fig 1. Percentage of generated studies for which a continuity correction is applied

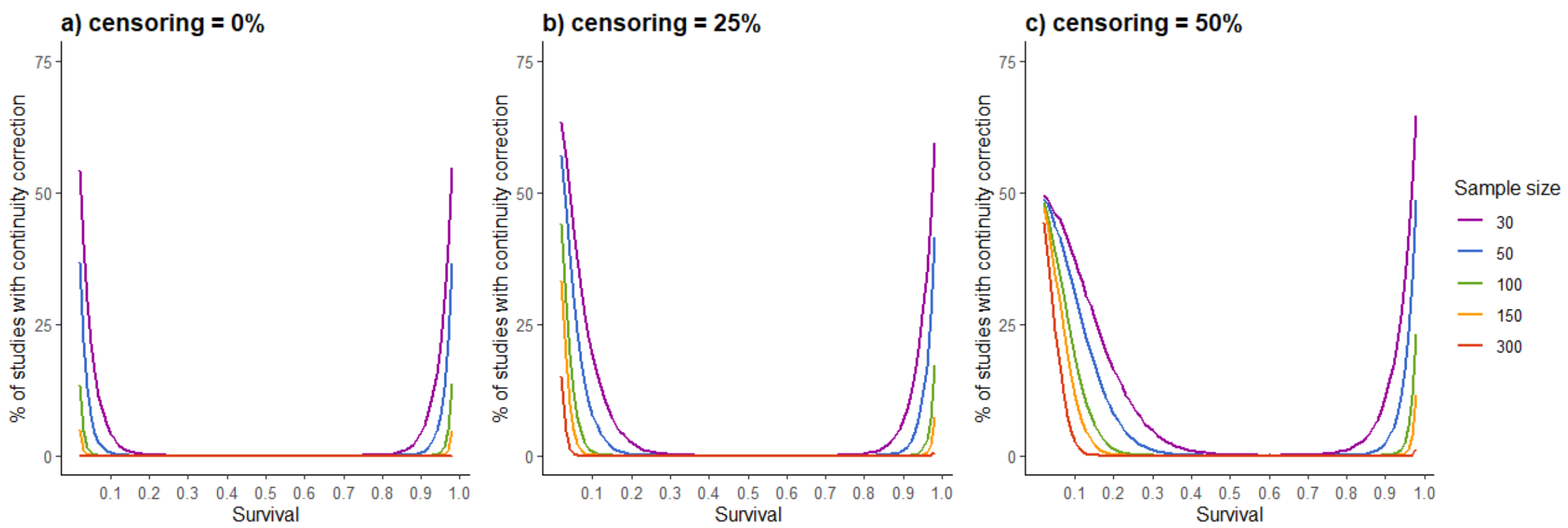

References

1. Nemes S, Gustavsson A, Taib Z. Variance Inflation Due to Censoring in Survival Probability Estimates. Statistica. 2020;80(4):395–412.
