# Supplementary material for: Extracting standard error from the 95% confidence interval of a survival
Source: PLoS One. 2026 Aug 4;21(8):e0355291. doi: 10.1371/journal.pone.0355291 (PMC13436750; doi:10.1371/journal.pone.0355291)

Fig 1. Extraction error for survival and 95%CIs reported with 4 decimal places

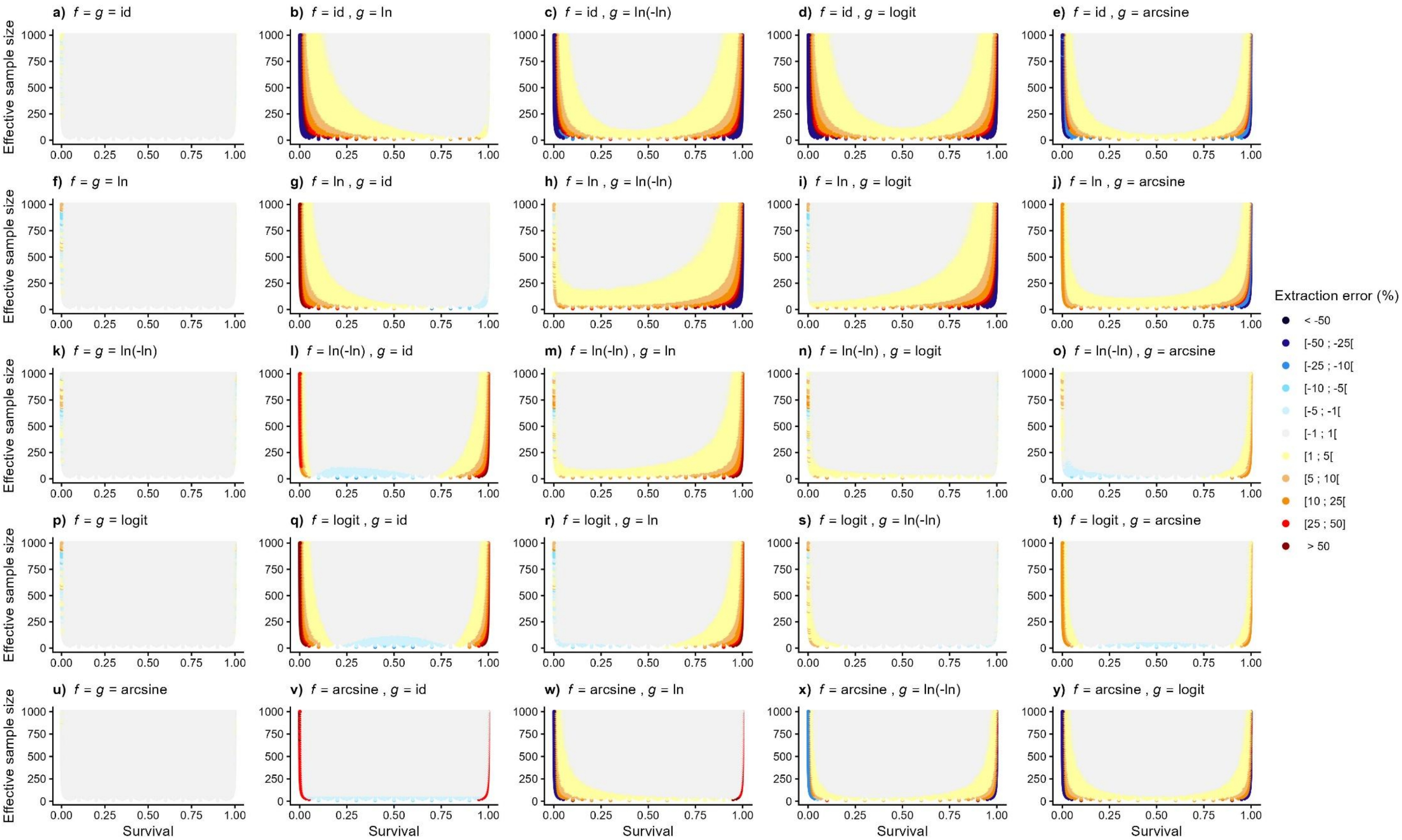

Fig 2. Extraction error for survival and 95%CIs reported with 3 decimal places

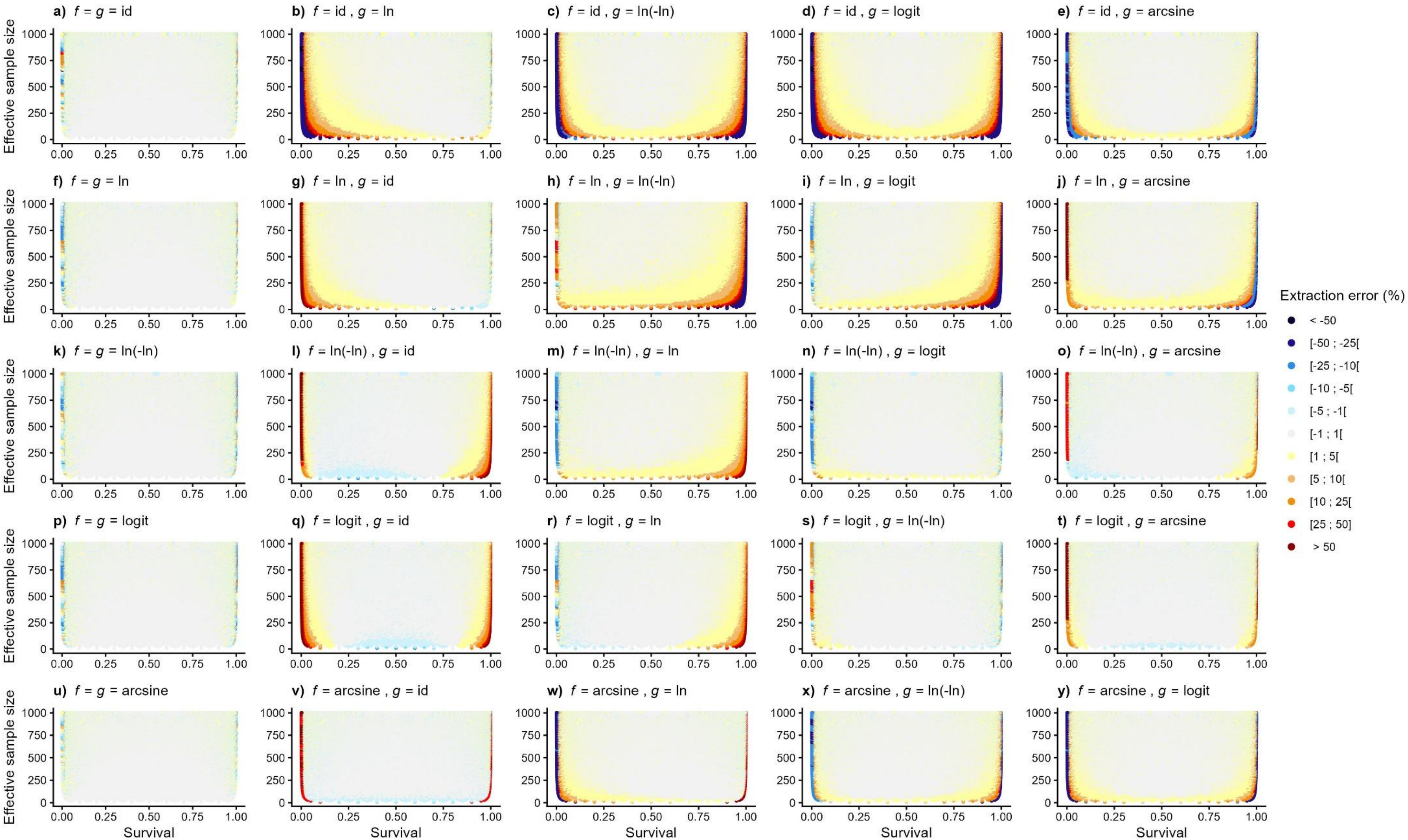

Fig 3. Extraction error for survival and 95%CIs reported with 2 decimal places

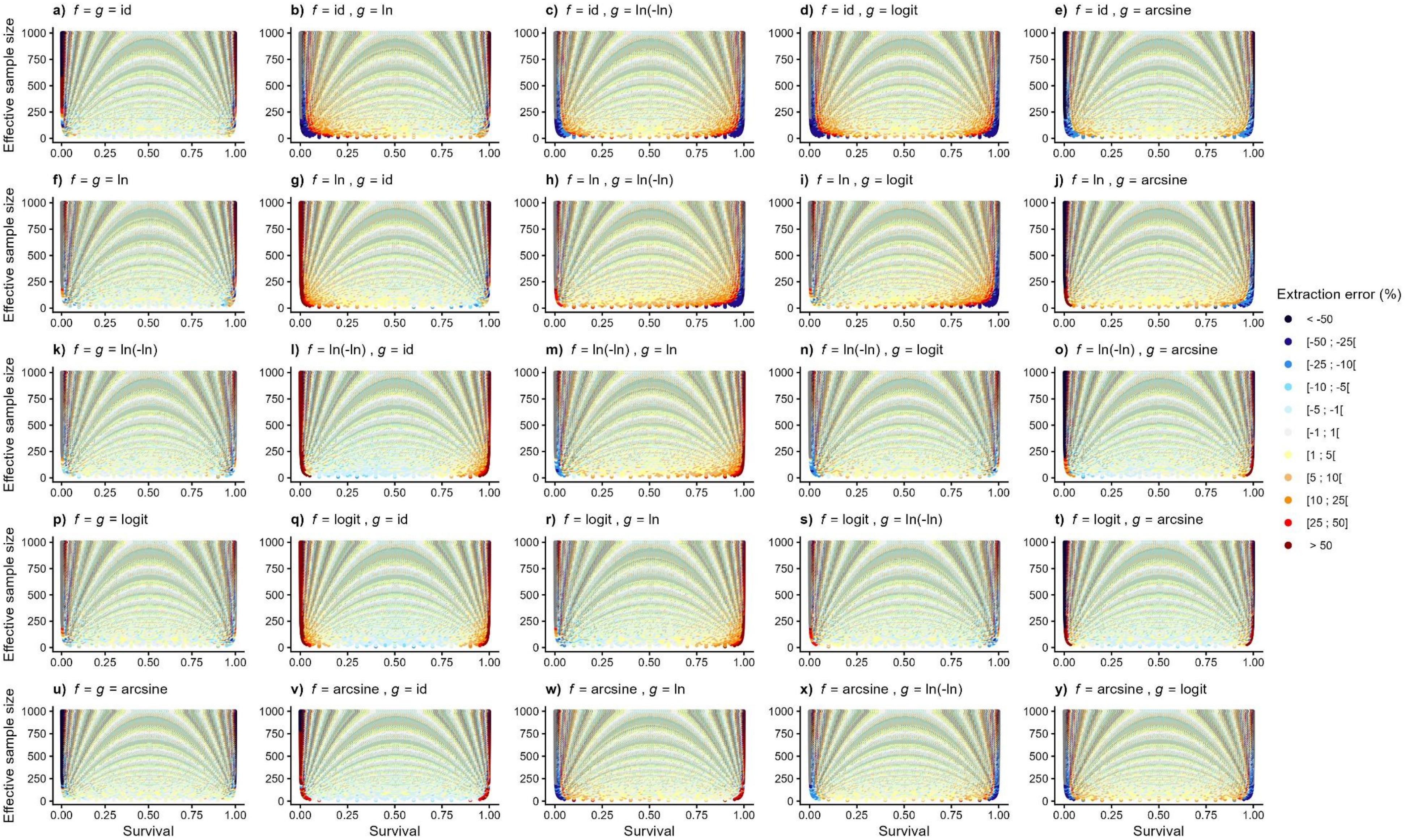

Supplement: Supplementary file 7 [file pone.0355291.s007.pdf]
