# Supplementary material for: Extracting standard error from the 95% confidence interval of a survival
Source: PLoS One. 2026 Aug 4;21(8):e0355291. doi: 10.1371/journal.pone.0355291 (PMC13436750; doi:10.1371/journal.pone.0355291)

Fig 1. Performance of the LRA approach in identifying *f*.

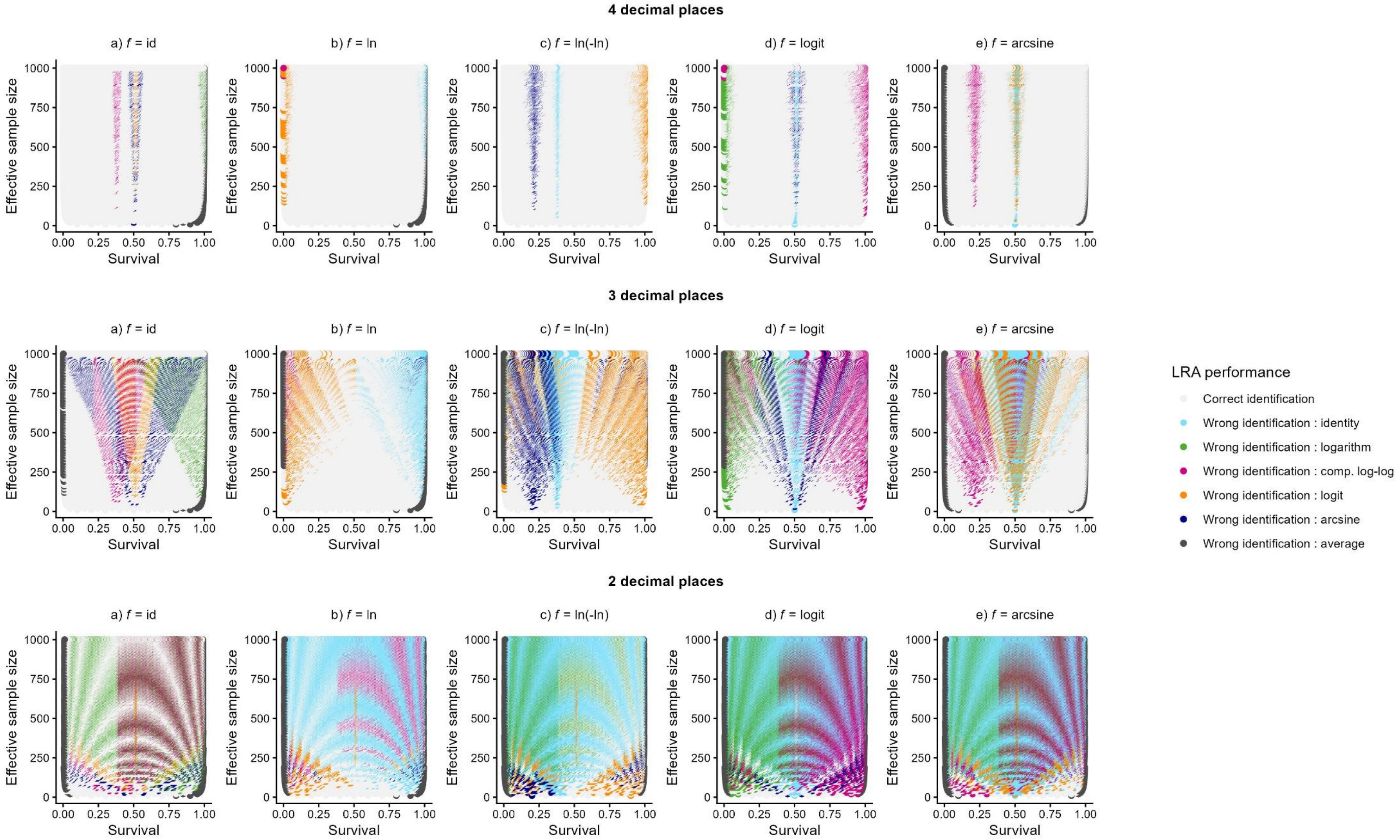

Fig 2. Extraction error when LRA approach is used to identify *f*.

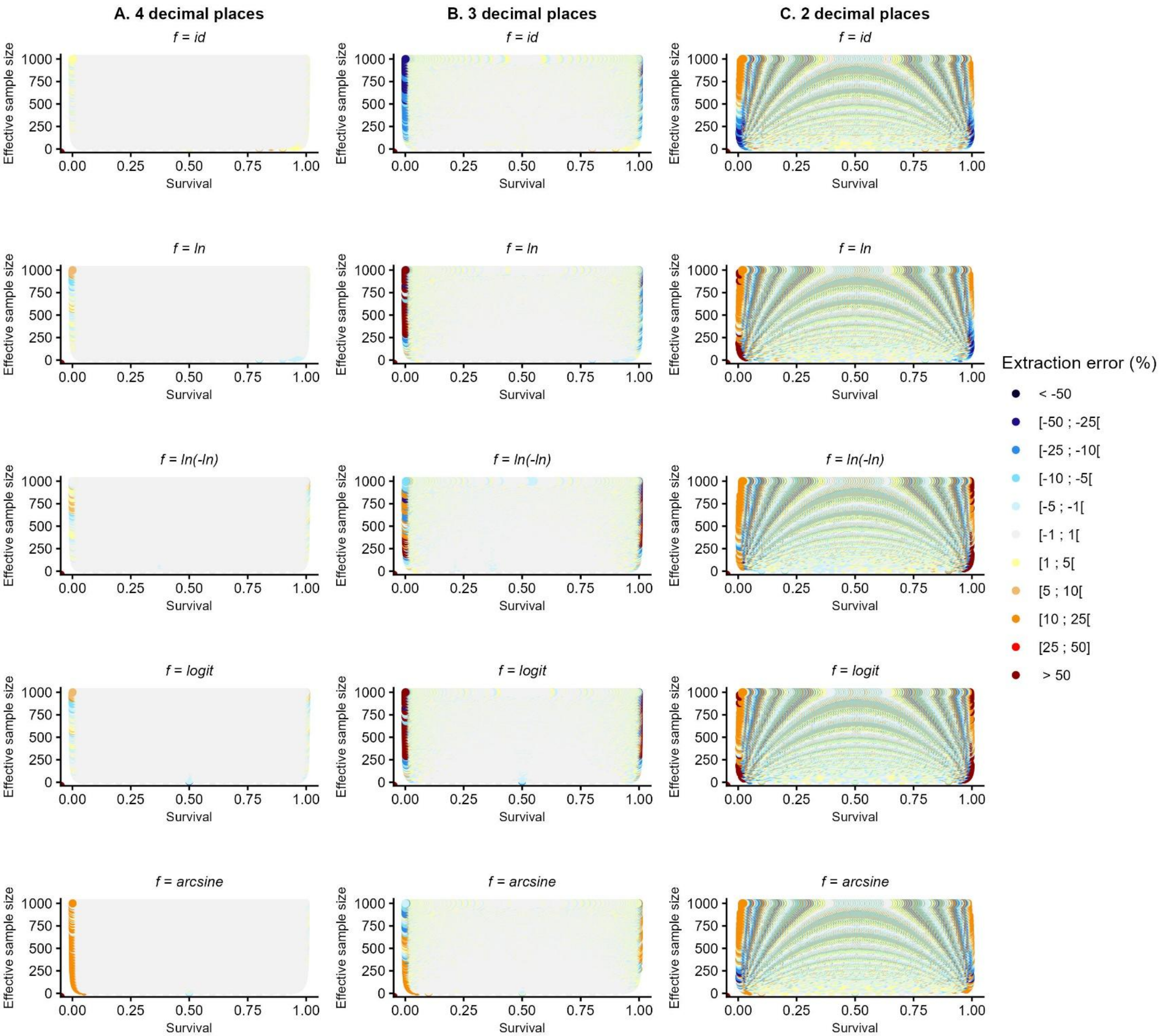

Supplement: Supplementary file 8 [file pone.0355291.s008.pdf]
