# Supplementary material for: Extracting standard error from the 95% confidence interval of a survival
Source: PLoS One. 2026 Aug 4;21(8):e0355291. doi: 10.1371/journal.pone.0355291 (PMC13436750; doi:10.1371/journal.pone.0355291)

Fig 1. Difference with correct pooled survival for a 0% censoring rate.

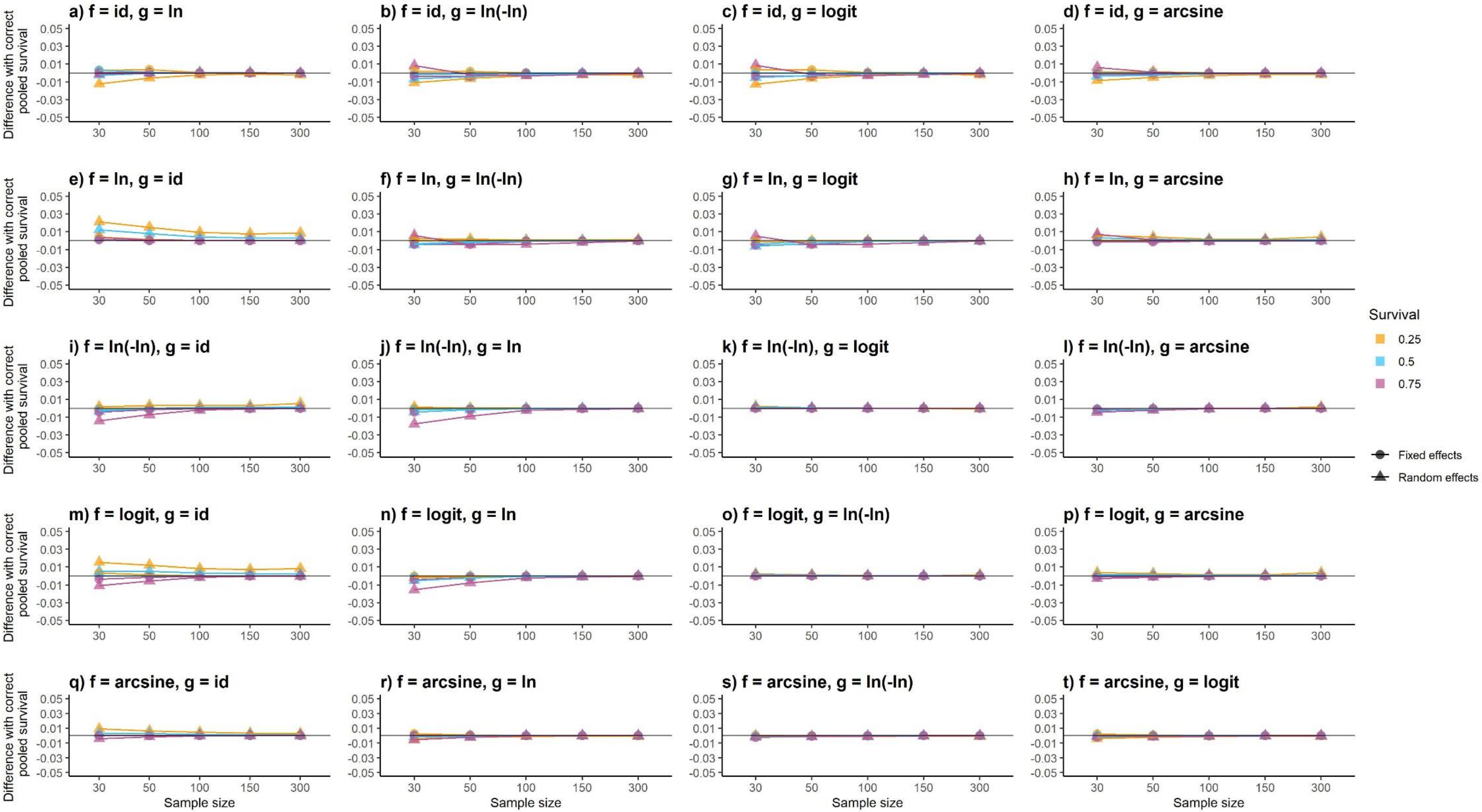

Fig 2. Difference with correct pooled survival for a 25% censoring rate.

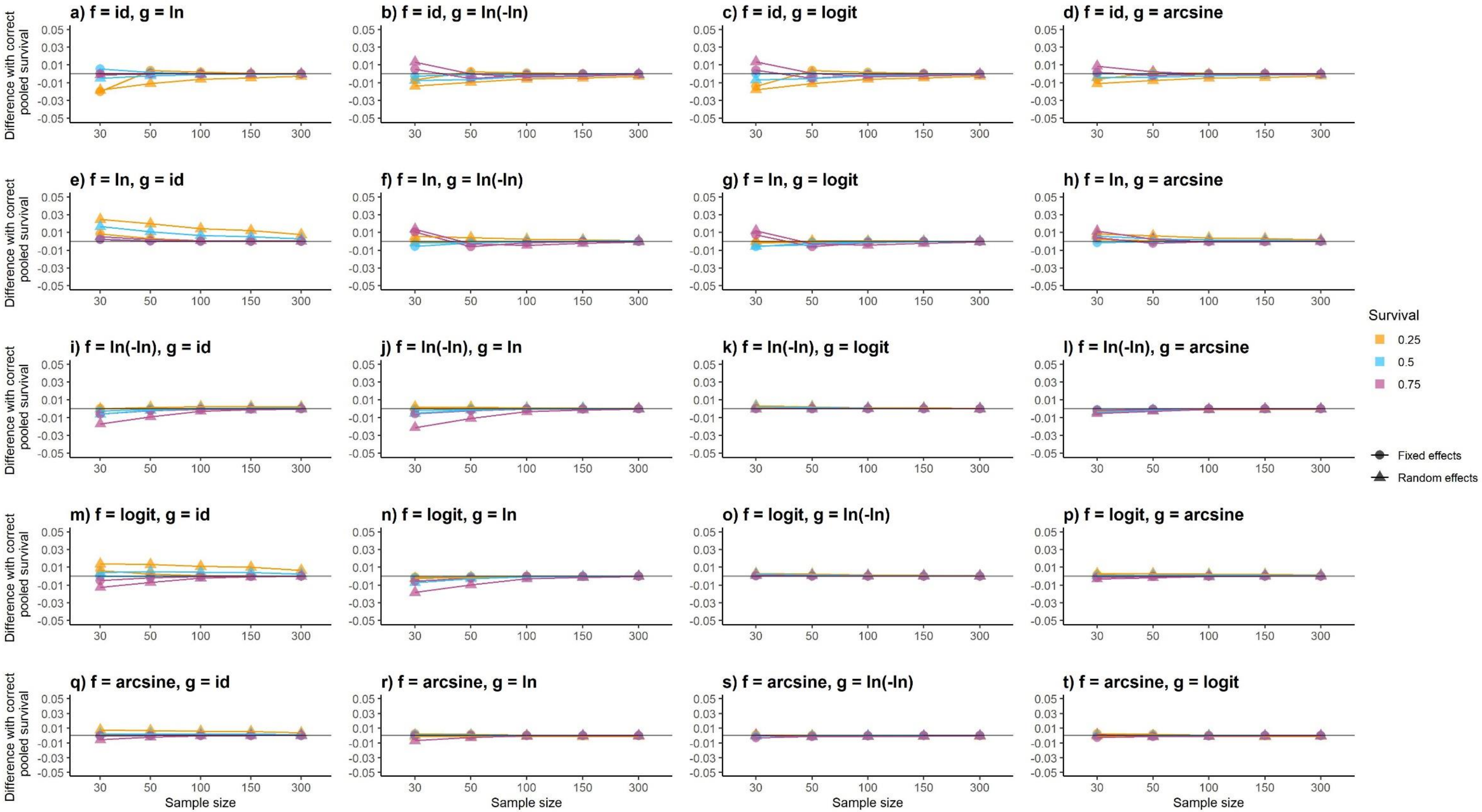

Fig 3. Difference with correct pooled survival for a 50% censoring rate.

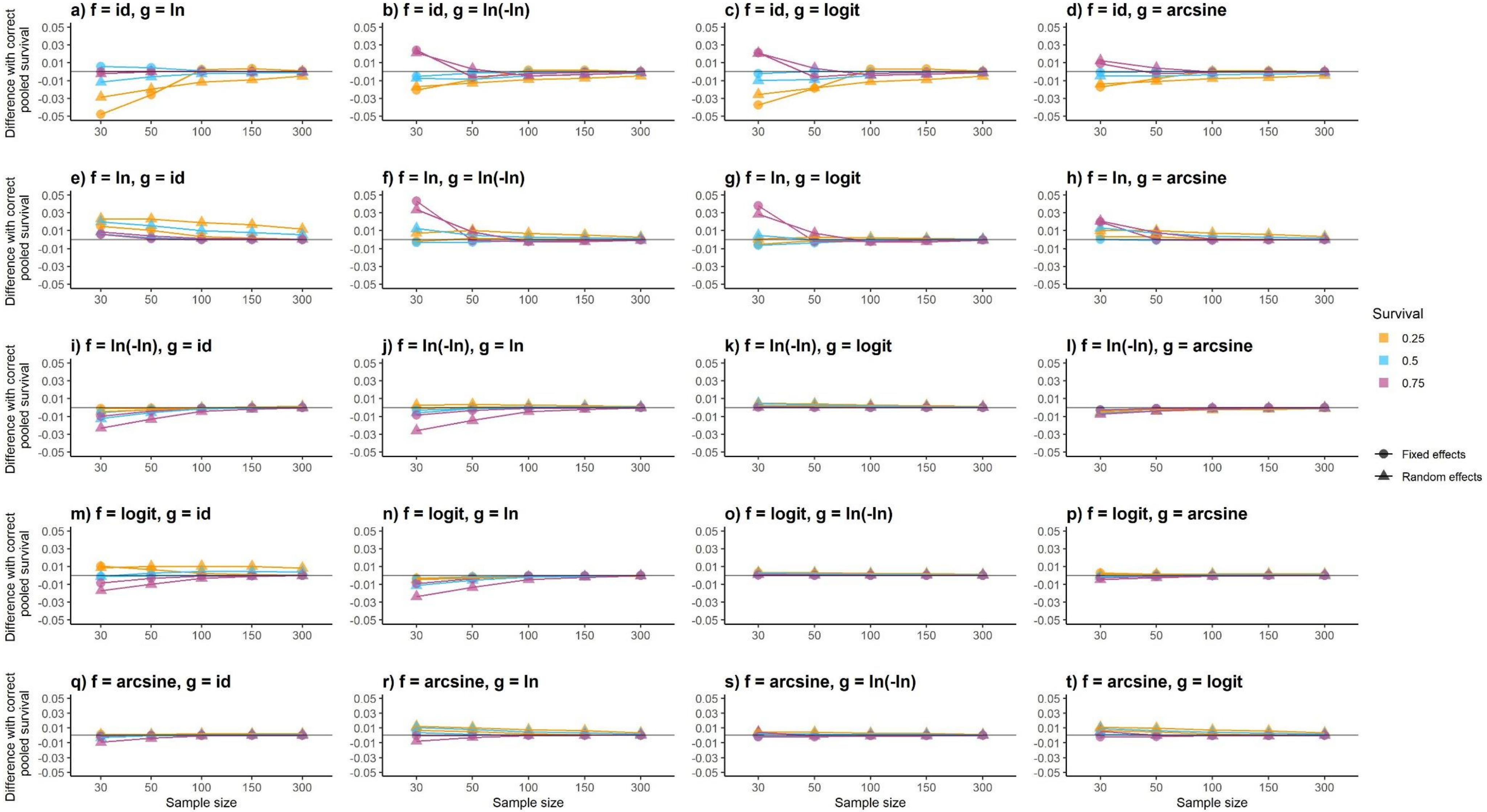

Fig 4. Difference in standard error for a 0% censoring rate.

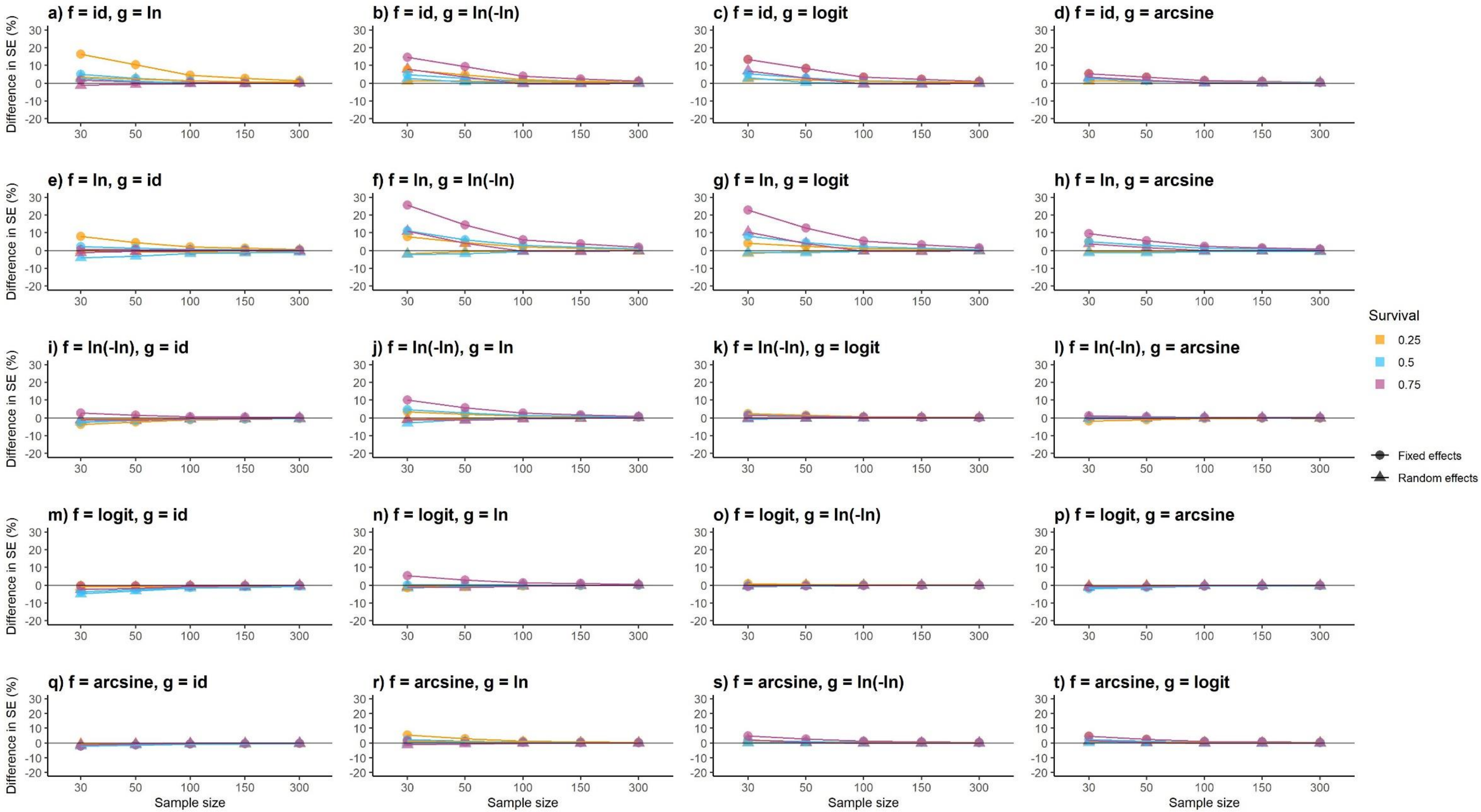

Fig 5. Difference in standard error for a 25% censoring rate.

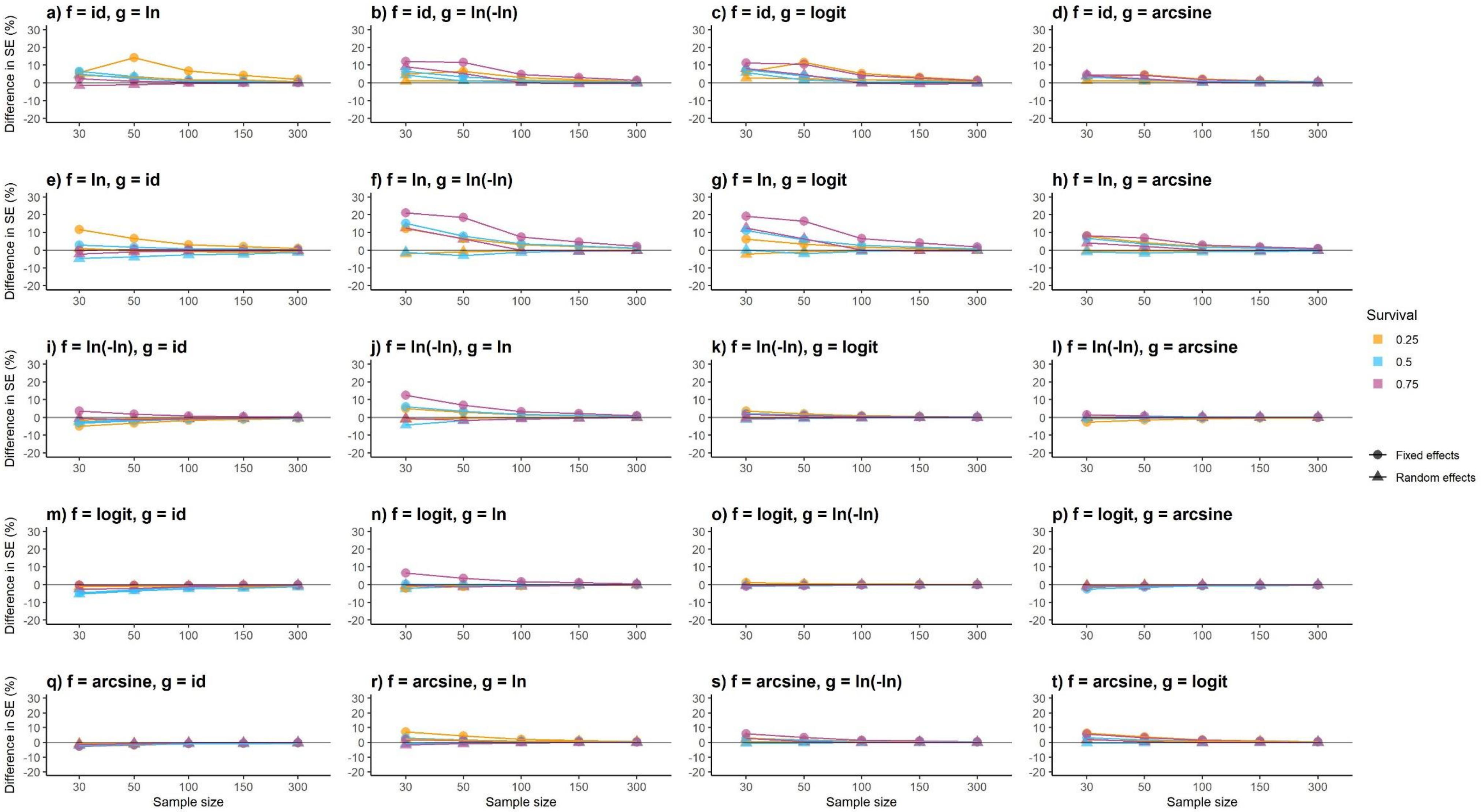

Fig 6. Difference in standard error for a 50% censoring rate.

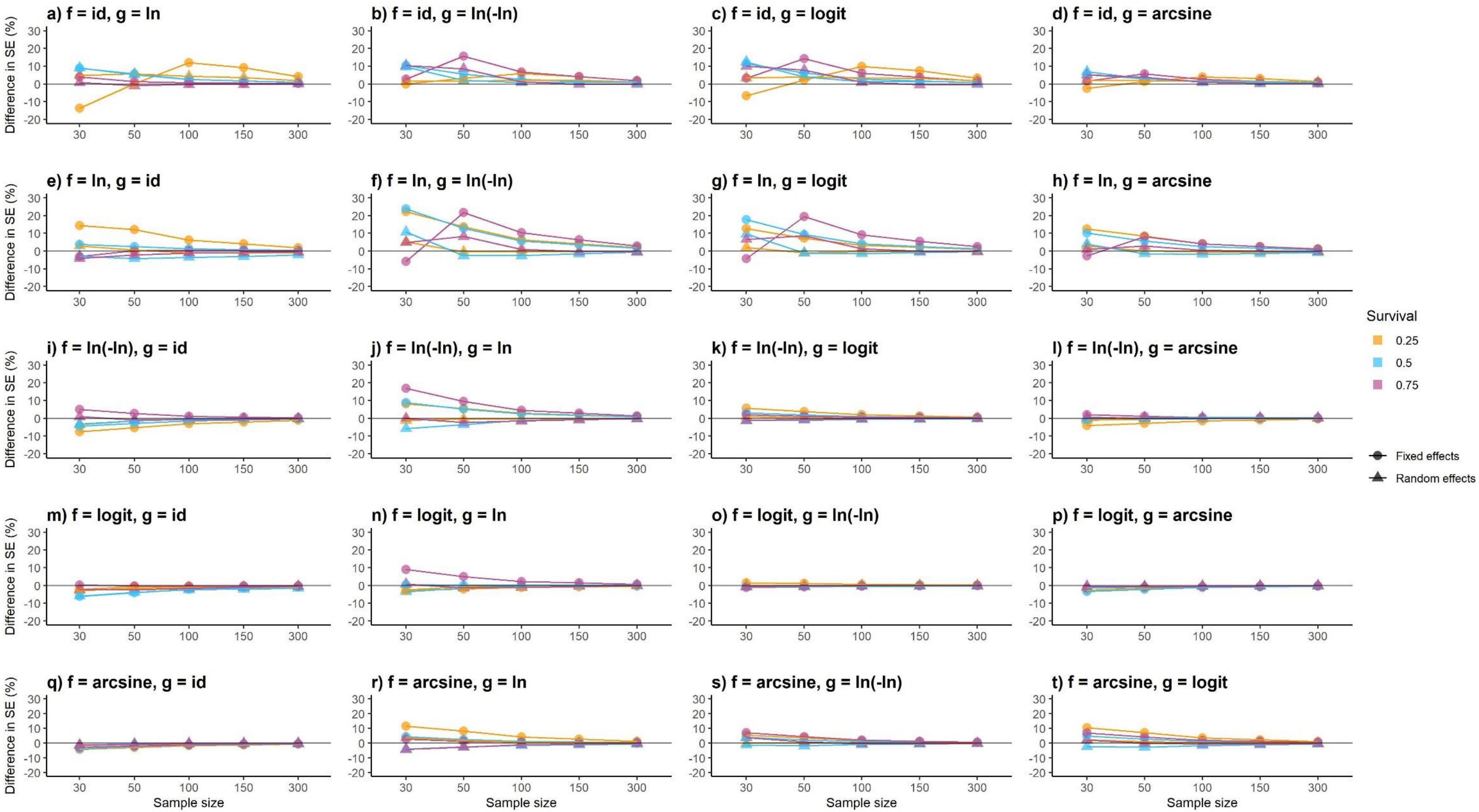

Fig 7. Difference with correct pooled survival and difference in standard error when LRA approach is used. The censoring rate is 0%.

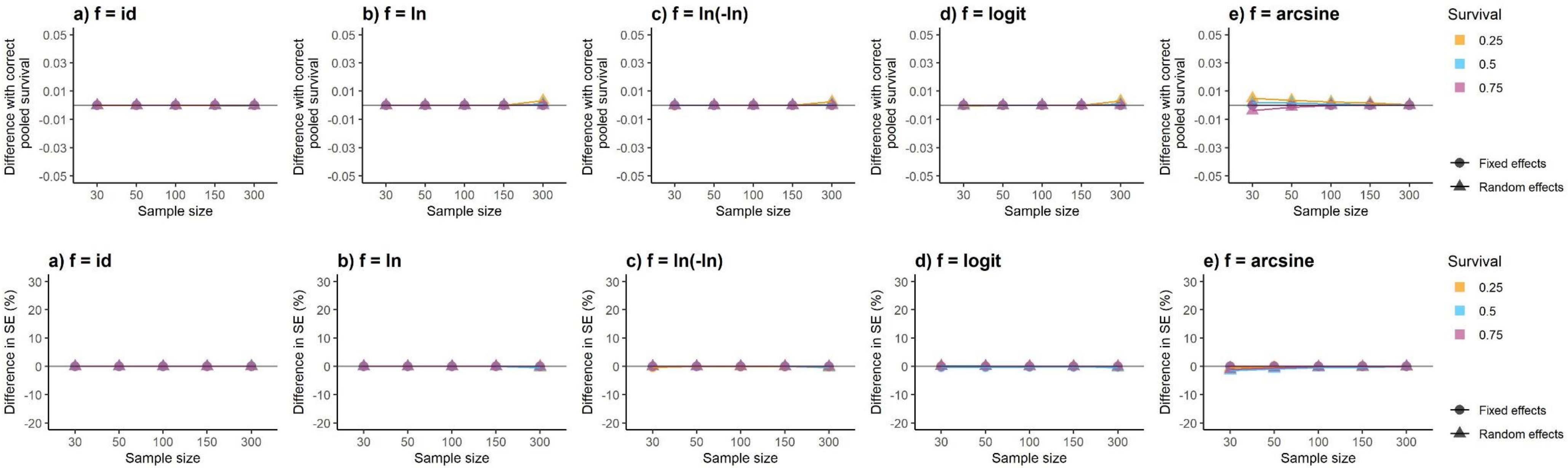

Fig 8. Difference with correct pooled survival and difference in standard error when LRA approach is used. The censoring rate is 25%.

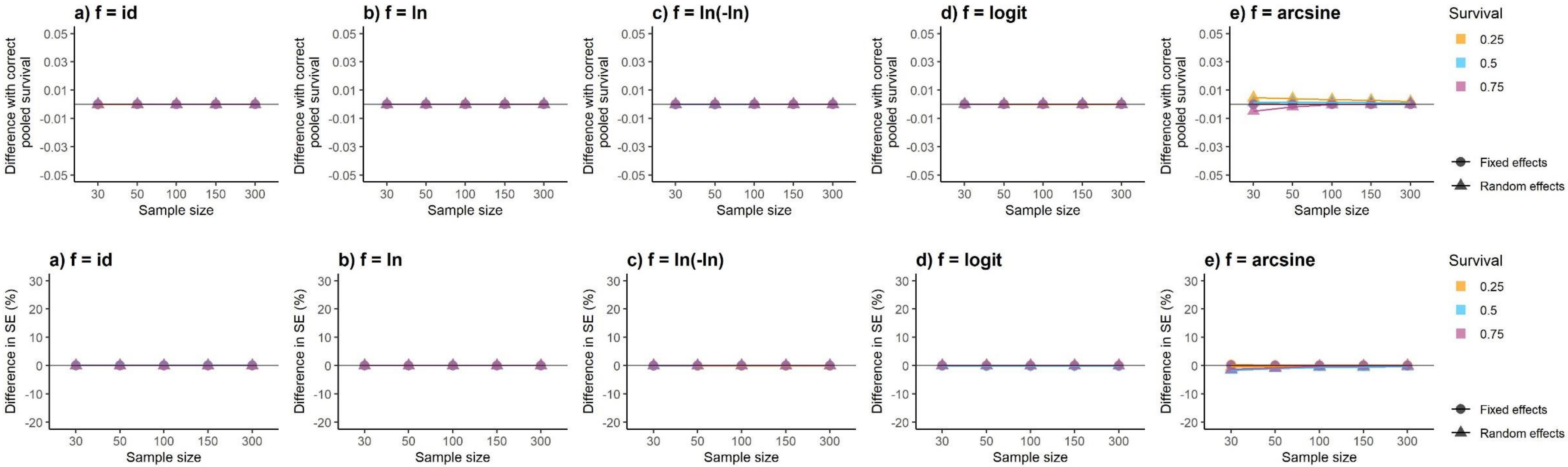

Fig 9. Difference with correct pooled survival and difference in standard error when LRA approach is used. The censoring rate is 50%.

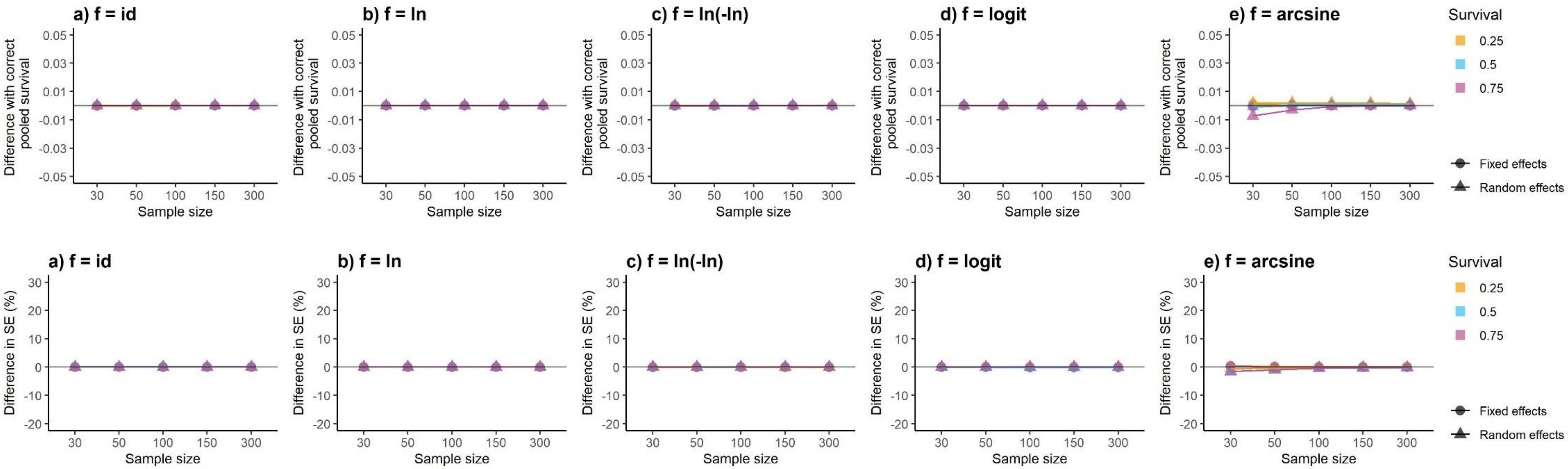

Supplement: Supplementary file 10 [file pone.0355291.s010.pdf]
