# Supplementary material for: Extracting standard error from the 95% confidence interval of a survival
Source: PLoS One. 2026 Aug 4;21(8):e0355291. doi: 10.1371/journal.pone.0355291 (PMC13436750; doi:10.1371/journal.pone.0355291)
Supplement: Supplementary file 12 [file pone.0355291.s012.pdf]

95%CIs of studies includes in the meta-analyses of the illustrative example.

		95%CI computed with				
Studies	Survival	*f(.)=id(.)*	*f(.)=ln(.)*	*f(.)=ln(-ln)*	*f(.)=logit(.)*	*f(.)=arcsine(.)*
1	0.111	0.000 to 0.256	0.030 to 0.410	0.019 to 0.298	0.028 to 0.352	0.012 to 0.292
2	0.711	0.541 to 0.881	0.559 to 0.903	0.503 to 0.844	0.518 to 0.849	0.530 to 0.862
3	0.093	0.000 to 0.213	0.026 to 0.337	0.017 to 0.251	0.024 to 0.298	0.011 to 0.244
4	0.361	0.175 to 0.548	0.215 to 0.605	0.184 to 0.541	0.201 to 0.559	0.190 to 0.553
5	0.277	0.177 to 0.376	0.193 to 0.396	0.183 to 0.378	0.189 to 0.386	0.184 to 0.380
